# Student engagement and self-regulated learning across standard and AI-enhanced flipped learning: a within-subjects longitudinal study

**DOI:** 10.3389/fpsyg.2026.1936442

**Published:** 2026-08-19

**Authors:** Chamintsetseg Myagmarkhorloo, Bat-Uchral Ganzorigt, Anita Habók

**Affiliations:** 1 Doctoral School of Education, University of Szeged, Szeged, Hungary; 2 Department of British and American Studies, National University of Mongolia, Ulaanbaatar, Mongolia; 3 Department of European Studies, National University of Mongolia, Ulaanbaatar, Mongolia; 4 Institute of Education, University of Szeged, Szeged, Hungary; 5 MTA-SZTE Digital Learning Technologies Research Group, Hungary

**Keywords:** flipped classroom, generative artificial intelligence, higher education, longitudinal study, Mongolia, self-regulated learning, student engagement

## Abstract

**Introduction:**

Although AI is increasingly being integrated into flipped classrooms, little is known about how student engagement and self-regulated learning (SRL) develop across extended implementations, particularly in underrepresented higher education contexts. This study examined developmental trajectories of student engagement and SRL across a sequential implementation comprising a standard flipped phase followed by a progressively scaffolded AI-enhanced phase, representing one of the earliest longitudinal investigations of AI-enhanced flipped learning in a Central Asian higher education context.

**Methods:**

Using a within-subjects longitudinal design, 211 undergraduate students at a public university in Mongolia were followed across four measurement occasions (Weeks 1, 6, 11, and 15) within a 16-week course, with quantitative data collected across 15 weeks of instruction. Engagement and SRL were assessed across a standard flipped phase (Weeks 1–6) and a progressively scaffolded AI-enhanced phase (Weeks 7–15). Metacognitive self-regulation was examined as a potential mediator of the longitudinal relationship between agentic engagement and academic achievement using a cross-lagged panel model (Mplus 8.3, ML estimator, 1,000 bootstrap samples).

**Results:**

Linear mixed-model analyses revealed significant trajectories for all engagement and SRL subscales (all *p* ≤ 0.004, η^2^_*p*_ = 0.021–0.111). All six SRL subscales remained stable during the standard flipped phase before increasing significantly during the AI-enhanced phase. Agentic engagement demonstrated the strongest engagement trajectory (η^2^_*p*_ = 0.103), whereas metacognitive self-regulation showed the largest SRL effect (η^2^_*p*_ = 0.111), with the greatest gains observed during the W11-W15 interval. Cross-lagged analyses identified a significant longitudinal association between agentic engagement at Week 1 and metacognitive self-regulation at Week 6 (β = 0.187, *p* = 0.005), whereas the indirect effect on academic achievement was not significant (β = 0.002, 95% BC CI [−0.028, 0.035]).

**Discussion:**

The findings describe distinct longitudinal trajectories of engagement and SRL across sequential instructional phases and support a developmental interpretation in which standard flipped instruction and progressively scaffolded AI integration may play distinct roles in supporting student engagement and SRL. This interpretation warrants evaluation in future controlled longitudinal studies.

## 1 Introduction

Flipped classrooms, defined as instructional designs in which content delivery moves outside class time to maximize opportunities for active learning during class, have been widely adopted in higher education (Akçayir and Akçayir, 2018; Låg and Sæle, 2019). A primary aim of this pedagogical approach is to foster student engagement and self-regulated learning (SRL). By shifting foundational content acquisition outside the classroom, flipped learning enables class time to be devoted to collaborative, problem-based, and application-oriented activities that promote deeper learning (Bergmann and Sams, 2012; Myagmarkhorloo and Habók, 2026). Recent experimental evidence further suggests that flipped learning can strengthen students' self-regulation and, when combined with synchronous online discussions, enhance behavioral and cognitive engagement (Yorganci, 2025). More recently, artificial intelligence (AI) has emerged as a scaffolding tool with the potential to further enhance flipped pedagogy by supporting autonomous learners capable of directing their own learning with AI assistance (Kasneci et al., 2023; Lee et al., 2024; Ouyang and Jiao, 2021). However, these benefits are not automatic: current discourse often conflates the physical relocation of content delivery with the pedagogical design attributes, such as structured AI scaffolding, that drive meaningful change. In one early pilot implementation, graduate students attributed skill gains more strongly to the flipped format itself than to chatbot use, prompting calls for more refined integration strategies that promote critical and responsible AI use (Tang et al., 2025).

Despite growing interest in AI-enhanced flipped classrooms, three critical gaps remain. First, evidence for engagement and SRL trajectories across extended instructional designs remains scarce; most studies rely on pre-post designs at single timepoints (Panadero, 2017; Zawacki-Richter et al., 2019). Second, the pathway from agentic engagement to metacognitive self-regulation, while theoretically well-motivated, has received limited empirical attention across instructional phases; existing cross-lagged panel model (CLPM) studies of agentic engagement have traced its reciprocal effects on autonomy-supportive teaching (Jang et al., 2024; Matos et al., 2018) but not its subsequent development of self-regulatory strategies. Third, the generalizability of AI-enhanced flipped learning models to non-Western, non-English-speaking higher education contexts remains largely unexamined; even foundational agentic engagement research, while extending to university samples in contexts such as Peru (Matos et al., 2018), has not been tested within AI-enhanced instructional designs in Central Asian or comparable settings.

This study addresses all three gaps through a 16-week within-subjects longitudinal investigation at one public university in Mongolia (*N* = 293 enrolled), implemented as a standard flipped phase (Weeks 1–6) with a three-phase progressive AI scaffolding intervention using Generative AI (GenAI) tools (Weeks 7–15: Guided, Semi-Guided, and Self-Directed), and concluding with reflective essay collection in Week 16. This design enabled the examination of engagement and SRL trajectories across a sequential implementation consisting of a standard flipped phase followed by a progressively scaffolded AI-enhanced phase.

Three complementary theoretical frameworks orient the study: Zimmerman's (2000) cyclical model of self-regulated learning, Chi and Wylie's (2014) ICAP framework, and Reeve and Tseng's (2011) construct of agentic engagement. Taken together, these frameworks provide the theoretical rationale for interpreting the observed longitudinal trajectories.

Four research questions guided the study:

1. How do student engagement trajectories evolve across the transition from standard to AI-enhanced flipped instruction?
2. How do self-regulated learning trajectories evolve across the transition from standard to AI-enhanced flipped instruction?
3. At which instructional transitions are the largest changes in engagement and SRL observed?
4. Does metacognitive self-regulation mediate the longitudinal relationship between agentic engagement and academic achievement?

## 2 Theoretical framework and literature

### 2.1 Student engagement in flipped classrooms

Student engagement is a multidimensional construct encompassing behavioral, emotional, cognitive, and agentic dimensions (Fredricks et al., 2004; Reeve and Tseng's, 2011). From a theoretical perspective, flipped classrooms provide a learning environment that supports these dimensions by reallocating classroom time from passive instruction to interactive, student-centered learning, thereby creating greater opportunities for active participation, higher-order thinking, collaboration, and student agency (Bergmann and Sams, 2012; Myagmarkhorloo and Habók, 2026). However, the effectiveness of this approach depends on factors such as students' preparation for class, technology access, and instructional design, which can influence the extent to which engagement is realized (Baig and Yadegaridehkordi, 2023). Meta-analytic evidence supports modest positive effects of flipped instruction on academic outcomes in higher education (Van Alten et al., 2019), a pattern broadly consistent with findings at the K-12 level (Zhu, 2021), though engagement effects specifically are more nuanced.

Låg and Sæle (2019) found only small effects of flipped instruction on learning, pass rates, and student satisfaction relative to traditional teaching, with effects likely attenuated by publication bias, suggesting that engagement-related benefits are not guaranteed by format alone. Akçayir and Akçayir (2018) similarly identified inadequate student preparation for out-of-class activities as the most commonly reported challenge undermining flipped classroom effectiveness, pointing to implementation quality rather than the format itself as the key constraint. Engagement trajectories are further moderated by pedagogical design quality: a PRISMA-guided systematic review of 25 higher education flipped classroom studies found that behavioral and cognitive engagement were most consistently enhanced when designs incorporated structured in-class active learning and formative accountability mechanisms, while emotional and agentic engagement showed more variable trajectories (Myagmarkhorloo and Habók, 2026). A recent preprint study using causal machine learning methods similarly found that flipped formats can improve student self-conception and reduce procrastination even when exam scores do not significantly change, underscoring that engagement and achievement are distinct outcome dimensions requiring separate measurement strategies (Czarnowske et al., 2025). These accumulated findings suggest that engagement trajectories in flipped classrooms are shaped less by instructional format and more by the scaffolding mechanisms embedded within each phase.

When GenAI is layered onto flipped designs, its effects on each engagement dimension become theoretically distinct. A systematic review and meta-analysis of 17 empirical studies found that ChatGPT-based learning produces medium effect sizes on behavioral and emotional engagement and a large effect size on cognitive engagement relative to non-ChatGPT learning, with ChatGPT functioning primarily through personalized tutoring, technical assistance, and content generation and collaboration (Heung and Chiu, 2025). Taken together with evidence that GenAI support creates a non-judgmental environment that reduces anxiety and builds confidence and wellbeing (Liu et al., 2026), this suggests AI-enhanced flipped contexts may address some of the emotional and behavioral disruption associated with standard flipped formats by reducing the evaluative pressure students associate with seeking help in front of peers.

Chi and Wylie's (2014) ICAP framework, introduced above, further predicts differential engagement outcomes across instructional phases, a pattern supported both by their original validation studies and by a large meta-analysis of college classroom outcomes (Freeman et al., 2014). Within the instructional design adopted in this study, the standard flipped phase aligns primarily with the Active mode, in which students manipulate pre-recorded content and complete structured learning activities with relatively limited opportunities for knowledge generation or dialogic co-construction. By contrast, the AI-enhanced phase was designed to promote Constructive and Interactive engagement by requiring students to formulate prompts, evaluate AI-generated responses, and iteratively refine their understanding through dialogue with AI. The ICAP framework therefore provides the theoretical basis for expecting differential engagement and SRL trajectories across instructional phases, a prediction examined in the present longitudinal design.

Recent work has begun applying ICAP directly to GenAI-mediated learning. Gao et al. (2024) combined ICAP with self-determination theory to show that ChatGPT integration enhances academic performance partly through motivational pathways, including learning desires, self-efficacy, and future beliefs. At the behavioral level, recent interaction-based research suggests that GenAI-mediated learning outcomes vary by engagement profile, with passive or retrieval-oriented chatbot use associated with weaker performance and collaborative, idea-developing partnerships with AI associated with stronger outcomes, reinforcing the ICAP prediction that Constructive and Interactive engagement, not mere AI output consumption, drives deeper learning (Oliveira et al., 2025).

### 2.2 Self-regulated learning and AI scaffolding

Self-regulated learning encompasses the cognitive, metacognitive, and motivational strategies students use to plan, monitor, and evaluate their own learning (Pintrich, 2004; Zimmerman's, 2000). The MSLQ framework (Pintrich et al., 1991) operationalizes SRL through six strategy subscales: rehearsal, elaboration, organization, critical thinking, metacognitive self-regulation, and help-seeking. Research consistently shows that metacognitive self-regulation is the most predictive subscale for academic achievement (Panadero, 2017). A 5-year systematic review of blended learning further confirms that resource management, motivational beliefs, and metacognitive knowledge carry significant positive impacts on higher education learning outcomes, positioning SRL strategies as central rather than peripheral to blended instructional success (Luo and Zhou, 2024).

Zimmerman's (2000) cyclical SRL model, introduced above, comprises forethought (goal-setting and planning), performance (strategy execution and monitoring), and self-reflection (evaluation and adjustment), with self-reflection feeding directly into the next cycle's forethought to generate a developmental spiral. Applied to this study, the standard flipped phase (W1–W6) is theorized to operate primarily at the forethought level, building content knowledge and motivational orientation without the on-demand dialogic feedback needed to activate performance-phase monitoring and self-reflection. Based on Zimmerman's framework, the AI-enhanced phase (W7–W15) was hypothesized to provide conditions that could better support the full cycle: students plan queries (forethought), monitor and evaluate AI responses (performance), and revise prompting strategies accordingly (self-reflection). The progressive Guided, Semi-Guided, and Self-Directed scaffolding design is intended to release this regulatory responsibility incrementally, providing the theoretical rationale for expecting that SRL strategy use will remain stable during the flipped phase and increase during the AI-enhanced phase, the dormant-then-activated pattern empirically investigated here.

GenAI tools introduce a qualitatively new form of SRL scaffolding by providing on-demand, personalized, dialogic feedback that instructors cannot replicate at scale. This progressive design parallels what Ouyang and Jiao (2021) describe as the field's broader shift from AI-directed to AI-empowered education, in which learners move from being recipients of AI services to leaders who take agency over their own learning. Kasneci et al. (2023) similarly argue that realizing the educational benefits of large language models depends on students developing critical thinking and fact-checking competencies rather than passively accepting AI output, underscoring that the impact of AI on learning is contingent on how students engage with it rather than on access alone. Empirically, the Guidance-based ChatGPT-assisted Learning Aid (GCLA; Lee et al., 2024) demonstrates, via a randomized controlled trial with undergraduate chemistry students, that providing hints rather than direct answers enhances SRL, higher-order thinking, and knowledge construction compared to traditional ChatGPT use.

Convergent evidence reinforces this scaffolding-dependent pattern. A meta-analysis of 35 studies spanning a decade of AI-SRL research found that generative AI produces substantially stronger SRL effects than earlier rule-based or adaptive systems, particularly during the performance phase, though these effects depend heavily on instructional design and intervention duration, pointing to a “performance-competence divide” where AI's capacity to scaffold immediate performance currently outpaces its ability to build durable self-regulatory competence (Xu et al., 2026). Comparing ChatGPT-based “SRLbots” with rule-based chatbots, Ng et al. (2024) similarly found stronger knowledge acquisition, behavioral engagement, and motivation in the generative AI condition, attributed to personalized, flexible feedback. Acceptance is itself intertwined with metacognitive SRL: Dahri et al. (2024) found that perceived AI intelligence and personal competency predict preservice teachers' intention to use ChatGPT for metacognitive activities, suggesting pre-existing dispositions shape how productively learners harness AI affordances. Finally, an SRL-integrated flipped classroom study found strong associations between SRL behaviors, engagement, and academic performance, with self-regulation, cognitive engagement, and emotional competencies clustering together, underscoring the value of integrating affective and social dimensions alongside cognitive regulation (Maiti and Priyaadharshini, 2026). Together, this evidence reinforces that scaffolding design, not AI access alone, is the critical variable shaping SRL outcomes.

### 2.3 Agentic engagement as a mechanism

Reeve and Tseng's (2011) agentic engagement construct, introduced above, is most strongly expressed in contexts offering student choice, dialogic interaction, and opportunities for self-initiated inquiry (Utami and Kurniawati, 2024), all features of the progressive AI scaffolding design employed here. In AI-assisted learning, agentic engagement may be uniquely activated because the AI interlocutor responds to student-initiated prompts, placing the student in a genuinely agentic role rather than a receptive one.

Theoretically, agentic engagement is the dimension most likely to foster metacognitive self-regulation, because formulating productive AI queries requires students to monitor their own understanding, identify gaps, and strategically seek clarification, the core forethought and performance processes in Zimmerman's (2000) cyclical SRL model. This theoretical perspective provides the rationale for examining whether baseline agentic engagement predicts subsequent metacognitive self-regulation across instructional phases using a cross-lagged panel model. Given the sequential within-subjects design, the study was intended to characterize longitudinal changes in engagement and SRL across successive instructional phases rather than to isolate the causal effects of AI-enhanced instruction.

## 3 Method

### 3.1 Research design

This study employed a within-subjects longitudinal design with four quantitative measurement occasions across 15 weeks of instruction: Week 1 (baseline), Week 6 (end of the standard flipped phase), Week 11 (midway through the AI-enhanced phase), and Week 15 (end of the AI-enhanced phase). The final week of the semester (Week 16) was reserved for reflective essay collection. The sequential design enabled the characterization of longitudinal changes in student engagement and self-regulated learning across successive instructional phases but was not intended to isolate the causal effects of AI-enhanced instruction. Table 1 presents the study design and measurement schedule.

**Table 1:** Study design and measurement schedule.

Week	Phase	Engagement scale	MSLQ-M	Achievement test	AI learning logs
1	Baseline	✓	✓	✓	—
6	End of standard flipped	✓	✓	✓	—
7–9	AI phase 1: guided	—	—	—	✓
10–12	AI phase 2: semi-guided	—	—	—	✓
11	Mid AI-enhanced phase	✓	✓	—	—
13–15	AI phase 3: self-directed	—	—	—	✓
15	End of AI-enhanced phase	✓	✓	✓	—
16	Reflective essays	—	—	—	—

MSLQ-M, Mongolian Motivated Strategies for Learning Questionnaire. AI learning Logs were submitted at the end of each AI phase rather than at a specific week. ✓ = administered; — = not administered.

### 3.2 Participants

Participants were undergraduate students enrolled in a semester-long elective course at a public university in the Mongolian capital during the Spring 2026 semester. A total of 293 students were registered across three course sections at the start of the semester. The course was delivered by the same instructor across all three sections using an identical syllabus, instructional materials, assessment schedule, and AI-supported learning activities. The same prompt templates, classroom activities, and assessment procedures were implemented in each section to ensure intervention consistency. Because the instructional design was identical across sections and students were not nested within different instructors or curricula, course section was not included as an analytical factor. During the semester, 35 students either withdrew from the course or failed to meet the university's minimum attendance requirement, leaving 258 active students. Among the 267 unique survey respondents, nine completed at least one survey wave before subsequently withdrawing from the course. Their responses were retained for the waves they had completed and contributed to the wave-level descriptive statistics, accounting for the difference between the 258 active participants and the 267 unique survey respondents.

After removing duplicate survey submissions, 267 unique respondents completed at least one survey wave (W1 = 264, W6 = 259, W11 = 237, W15 = 215). To ensure sufficient longitudinal coverage, participants were required to contribute data from at least 8 of the 10 study participation events, comprising four combined survey administrations (each including both the Engagement Scale and the MSLQ-M), three AI learning logs, and three achievement tests. Although the Engagement Scale and MSLQ-M are separate instruments, they were administered within a single questionnaire at each wave and were therefore counted as one participation event rather than two, yielding 10 participation events in total (4 survey sessions + 3 AI learning logs + 3 achievement tests). The ≥8-event criterion was selected to balance longitudinal data completeness with sample retention, ensuring sufficient repeated observations for reliable estimation of longitudinal trajectories and cross-lagged relationships while minimizing participant loss. Application of this criterion yielded the primary analytic sample of 211 students, which was used for all longitudinal inferential analyses unless otherwise specified. Of the 95 excluded participants, most had completed three or fewer participation events, indicating minimal overall engagement with the study rather than selective non-response to particular instruments. Attrition analyses indicated small but significant baseline differences in behavioral engagement, emotional engagement, and help-seeking between retained and excluded participants; no significant differences were observed for the remaining variables (Table 2; full results are presented in Supplementary Table S1). Sensitivity analyses using a less restrictive inclusion criterion (≥7 participation events; *n* = 251) produced substantively similar results, supporting the robustness of the primary findings.

**Table 2:** Baseline characteristics of retained and excluded participants.

Variable	Retained (*n* = 211)	Excluded (*n* = 95)	Test statistic	*p*
Age, M (SD)	20.1 (1.5)	19.8 (1.6)	*t* = 1.41 (d = 0.19)	0.160
Female, %	88.6	86.3	*χ^2^* = 0.37	0.545
GPA, M (SD)	3.06 (0.54)	3.01 (0.58)	*t* = 0.68 (d = 0.09)	0.499
Behavioral Engagement, M (SD)	4.13 (0.64)	3.84 (0.53)	*t* = 3.85 (d = 0.50)	**< 0.001**
Emotional Engagement, M (SD)	4.20 (0.72)	3.94 (0.73)	*t* = 2.31 (d = 0.36)	**0.022**
Help-Seeking, M (SD)	3.23 (1.03)	2.92 (0.96)	*t* = 2.37 (d = 0.30)	**0.019**

Independent-samples *t* tests with Welch's correction were used for continuous variables and a chi-square test for gender. No significant differences were observed for cognitive engagement, agentic engagement, the remaining self-regulated learning subscales, academic achievement, or year of study (all *p* > 0.05). Full attrition analyses are reported in Supplementary Table S1. Cohen's *d* values are shown in parentheses alongside *t* statistics. Bold values indicate statistically significant results (*p* < 0.05).

The final analytic sample had a mean age of 20.1 years (SD = 1.5; range = 17–24). Most participants were female (*n* = 187, 88.6%), reflecting the gender distribution typical of humanities programs in Mongolia. The majority were enrolled in the Humanities division of the School of Arts and Sciences (92.4%), followed by Social Sciences (3.3%), Business (1.9%), and other faculties (2.4%). Most students were in their second year of study (59.7%), followed by first-year (19.9%), third-year (11.8%), and fourth-year or above (8.5%). Mean GPA was 3.06 (SD = 0.54) on a four-point scale. Most participants had prior experience with AI tools: 79.1% had used AI occasionally, 13.3% reported regular use, and only 7.6% had no prior experience. At baseline, 55.0% reported using AI at least weekly, whereas 57.8% had never previously experienced flipped classroom instruction, indicating that the standard flipped phase represented a novel instructional approach for most students. Ethical approval was obtained from the Doctoral School of Education, University of Szeged (Approval No. 16/2025). Permission to conduct the study was granted by the host university and course instructors. Electronic informed consent was obtained from all participants before data collection, and no personally identifiable information was retained.

### 3.3 Instructional context

The study was conducted in a 16-week elective undergraduate course in the field of applied linguistics at a public university in Mongolia. The course was open to students from all faculties, although enrolment was predominantly from humanities programs.

#### 3.3.1 Standard flipped phase (Weeks 1–6)

During the standard flipped phase, students completed pre-class video lectures and assigned readings before attending class. Face-to-face sessions focused on discussion, collaborative problem-solving, and application of theoretical concepts through active learning activities. No AI tools were incorporated during this phase, allowing the initial instructional period to serve as the reference condition within the sequential design.

#### 3.3.2 AI-enhanced flipped phase (Weeks 7–15)

During the AI-enhanced phase, students integrated self-selected generative AI (GenAI) tools into their learning through a progressive three-stage scaffolding design that gradually transferred regulatory responsibility from instructor to learner.

#### 3.3.3 Guided phase (Weeks 7–9)

The instructor provided structured prompt templates each week that students used directly or with minor modifications. Prompts focused on activities such as explaining theoretical concepts, generating personalized study plans, and creating short quizzes. The purpose of this phase was to familiarize students with effective and responsible AI-supported learning practices.

#### 3.3.4 Semi-guided phase (Weeks 10–12)

Students adapted the instructor's prompts to suit their own learning needs, specifying the purpose, depth, and desired format of AI responses. This phase encouraged students to experiment with prompt engineering strategies aligned with their individual learning preferences while critically evaluating AI-generated outputs.

#### 3.3.5 Self-directed phase (Weeks 13–15)

Instructor-provided prompts were withdrawn. Instead, students received learning objectives and assessment criteria and independently determined when, how, and for what purposes AI would support their learning. Students were expected to formulate their own prompts, critically evaluate AI responses against course materials and other authoritative sources, identify inaccuracies or incomplete information, and integrate AI strategically into their study routines.

Students were free to select their preferred generative AI platform throughout the intervention. The most frequently reported systems were ChatGPT, Google Gemini, and Claude, while a smaller number of students also reported using DeepSeek, Microsoft Copilot, and other generative AI tools. Following each instructional phase, students completed structured AI learning logs documenting the primary AI tools used, learning purposes, prompt strategies, and reflections on the usefulness and limitations of AI support. In the present study, these logs were used solely to monitor intervention implementation and participant adherence; they were not analyzed as a source of qualitative data. As an implementation fidelity measure, 233 of the 258 active students (90.3%) submitted a Phase 1 log, 218 (84.5%) submitted a Phase 2 log, and 206 (79.8%) submitted a Phase 3 log. Among students submitting logs, reported AI use increased from 95.3% during Phase 1 to 98.5% during Phase 3. All three intervention phases were delivered by the same instructor using the same course structure and assessment procedures.

### 3.4 Instruments

All instruments were administered in Mongolian to ensure linguistic accessibility and measurement validity. Example items are presented in English translation. Both the Engagement Scale and the Mongolian version of the Motivated Strategies for Learning Questionnaire (MSLQ-M) were translated and culturally adapted following established cross-cultural adaptation guidelines (Beaton et al., 2000; Borsa et al., 2012; Klockar et al., 2024), emphasizing conceptual rather than literal equivalence. Comprehensive validation studies of both instruments have been submitted separately.

For the Engagement Scale, the adaptation involved independent forward translation, synthesis, back-translation, expert consensus, and approval of the pre-final version. Item-level refinements were subsequently informed by exploratory factor analysis and cognitive interview evidence to address statistical misfit, linguistic ambiguity, and contextual relevance within Mongolian higher education.

The MSLQ-M underwent a comparable multi-stage adaptation process comprising forward translation, back-translation, expert review, and cognitive evaluation with students. Minor linguistic revisions were made to improve clarity, and four items were substantially reworded following expert review. In particular, two Critical Thinking items were adapted to reflect culturally appropriate expressions of analytical thinking within teacher-directed higher education. Consequently, the Critical Thinking subscale represents a contextually adapted variant and should be interpreted accordingly. The complete bilingual item lists are available from the corresponding author upon reasonable request.

#### 3.4.1 Engagement scale

Student engagement was measured using a 20-item scale comprising four subscales: Behavioral Engagement (4 items; e.g., “*I pay close attention in class”*), Emotional Engagement (3 items; e.g., “*I am proud to study at this university”*), and Cognitive Engagement (8 items; e.g., “*When learning something new in class, I try to connect it with what I already know”*), adapted from the Student Engagement Scale (Lam et al., 2014), and Agentic Engagement (5 items; e.g., “*During class, I express my preferences and opinions”*), adapted from Reeve and Tseng's (2011). Items were rated on a 5-point Likert scale (1 = strongly disagree, 5 = strongly agree).

Internal consistency was good to excellent across all subscales and measurement occasions (Cronbach's α = 0.85–0.93; Table 3). Composite reliability (0.882–0.939) and AVE (0.580–0.837) indicated satisfactory convergent validity, and discriminant validity was supported according to the Fornell–Larcker criterion.

**Table 3:** Descriptive statistics and internal consistency for engagement and SRL subscales across four timepoints.

Subscale		W1 (baseline) *n* = 264		W6 (post-flipped) *n* = 259		W11 (Mid AI-enhanced phase) *n* = 237		W15 (End AI-enhanced phase) *n* = 215	
	*k*	M (SD)	α	M (SD)	α	M (SD)	α	M (SD)	α
Student engagement									
Emotional engagement	3	4.20 (0.72)	0.88	4.10 (0.82)	0.90	4.21 (0.80)	0.91	4.27 (0.76)	0.92
Behavioral engagement	4	4.13 (0.64)	0.85	3.98 (0.68)	0.86	4.04 (0.69)	0.89	4.21 (0.65)	0.87
Cognitive engagement	8	3.87 (0.67)	0.89	3.91 (0.68)	0.91	3.97 (0.69)	0.92	4.08 (0.67)	0.92
Agentic engagement	5	2.75 (0.96)	0.91	2.87 (1.06)	0.92	3.07 (1.03)	0.93	3.29 (1.13)	0.93
Self-regulated learning strategies (MSLQ-M)									
Rehearsal	4	3.65 (0.71)	0.67^+^	3.65 (0.71)	0.73	3.81 (0.71)	0.76	3.95 (0.69)	0.75
Elaboration	6	3.79 (0.67)	0.80	3.80 (0.66)	0.84	3.86 (0.68)	0.87	4.01 (0.62)	0.85
Organization	4	3.57 (0.80)	0.70	3.63 (0.75)	0.74	3.68 (0.76)	0.76	3.89 (0.72)	0.76
Critical thinking	5	3.73 (0.70)	0.81	3.76 (0.70)	0.84	3.87 (0.67)	0.85	3.95 (0.69)	0.85
Metacognitive self-regulation	12	3.48 (0.72)	0.89	3.53 (0.71)	0.90	3.64 (0.70)	0.91	3.83 (0.68)	0.91
Help-seeking	4	3.23 (1.03)	0.85	3.24 (1.02)	0.85	3.41 (1.00)	0.86	3.59 (0.95)	0.86

Means, standard deviations, and Cronbach's α were calculated from all respondents completing each survey wave (sample sizes shown in the column headings). Inferential longitudinal analyses were conducted using the restricted analytic sample (*N* = 211). ^+^ Cronbach's α < 0.70; interpret with caution.

A confirmatory factor analysis supported the hypothesized four-factor structure (CFI = 0.968, TLI = 0.963, RMSEA = 0.077, SRMR = 0.055). Standardized factor loadings ranged from 0.62 to 0.94 (all *p* < 0.001). Longitudinal measurement invariance analyses supported configural invariance (CFI = 0.905, RMSEA = 0.061) and metric invariance (ΔCFI = +0.007, ΔRMSEA = −0.003) across the four measurement occasions. Full scalar invariance was not supported (ΔCFI = −0.026); a partial scalar model was subsequently estimated but produced estimation instability (non-positive definite matrices for two items). Longitudinal trajectory comparisons were therefore interpreted under metric invariance, which is sufficient for observed-score analyses using linear mixed models (Vandenberg and Lance, 2000). Detailed CFA results, factor loadings, and invariance results are presented in Supplementary Tables S2, S4.

#### 3.4.2 Mongolian motivated strategies for learning questionnaire (MSLQ-M)

Self-regulated learning was assessed using the Mongolian adaptation of the Motivated Strategies for Learning Questionnaire (Pintrich et al., 1991), comprising positively worded items across six subscales: Rehearsal (4 items; e.g., “*When I study for this class, I practice saying the material to myself over and over”*), Elaboration (6 items; e.g., “*When reading for this class, I try to relate the material to what I already know”*), Organization (4 items; e.g., “*When I study the readings for this course, I outline the material to help me organize my thoughts”*), Critical Thinking (5 items; e.g., “*When a theory, interpretation, or conclusion is presented in class or in the readings, I try to decide if there is good supporting evidence”*), Metacognitive Self-Regulation (12 items; e.g., “*I ask myself questions to make sure I understand the material I have been studying in this class”*), and Help-Seeking (4 items; e.g., “*I ask the instructor to clarify concepts I don't understand well”*). Responses were recorded on the same five-point Likert scale.

Internal consistency ranged from Cronbach's α = 0.67–0.91 across subscales and measurement occasions (Table 3). Rehearsal showed the lowest reliability at baseline (α = 0.67), slightly below the conventional 0.70 criterion, a pattern commonly reported for this brief four-item subscale (Pintrich et al., 1991). Composite reliability ranged from 0.702 to 0.910. AVE values for four subscales fell below the conventional 0.50 criterion despite acceptable composite reliability, and discriminant validity was only partially supported according to the Fornell–Larcker criterion, reflecting the conceptual overlap among SRL strategies (Pintrich, 2004).

A confirmatory factor analysis supported the hypothesized six-factor structure (CFI = 0.945, TLI = 0.940, RMSEA = 0.057, SRMR = 0.062). Standardized factor loadings ranged from 0.53 to 0.89 (all *p* < 0.001). Longitudinal measurement invariance analyses supported configural invariance for all six subscales and metric invariance for most subscales; absolute model fit was below conventional thresholds for Elaboration, Critical Thinking, and Metacognitive Self-Regulation at the configural level, and findings for these subscales should therefore be interpreted with caution. Detailed CFA results, factor loadings, and invariance results are presented in Supplementary Tables S3, S5.

#### 3.4.3 Academic achievement

Academic achievement (ACHIE) was assessed using a researcher-developed 15-item multiple-choice test aligned with the course learning objectives. The assessment was administered at W1, W6, and W15 to evaluate knowledge acquisition across the intervention while minimizing participant burden. The same items were administered at each occasion, with item order randomized to reduce recall effects. Each correct response received one point, producing scores ranging from 0 to 15. For example, one item asked students to identify the theoretical framework most closely associated with the concept of language acquisition through comprehensible input. Although item order was randomized at each administration, repeated exposure to the same items may have introduced familiarity effects that partially inflated observed score gains independent of genuine knowledge acquisition; this limitation is discussed in Section 5.5.

Content validity was established through alignment with weekly learning objectives and review by both the course instructor and an independent subject specialist. Internal consistency was acceptable at each administration (KR-20 = 0.703, 0.702, and 0.740 for Weeks 1, 6, and 15, respectively). Item analysis identified two items with near-zero or negative corrected item-total correlations across waves (Item 10: *r* = −0.045 to −0.113; Item 11: *r* = −0.026 to 0.105), suggesting limited psychometric utility. These items were retained to maintain test-form consistency across the three administrations but should be noted as a limitation of the measure. One additional item (Item 13) showed ceiling-level difficulty (proportion correct >0.93 at all waves), contributing minimally to score variance. Item-level statistics including difficulty and discrimination coefficients for all 15 items across three administrations are provided in Supplementary Table S6.

### 3.5 Data analysis

Missing data were handled using likelihood-based estimation rather than listwise deletion. Linear mixed models (LMMs) were estimated in IBM SPSS Statistics Version 28 (IBM Corp., Armonk, NY, United States) using restricted maximum likelihood (REML), whereas the cross-lagged panel model (CLPM) was estimated in Mplus Version 8.3 (Muthén & Muthén, Los Angeles, CA, United States) using full information maximum likelihood (FIML; Enders, 2010). To evaluate potential attrition bias, baseline characteristics were compared between retained and excluded participants using independent-samples *t*-tests with Welch's correction and chi-square tests. A sensitivity analysis comparing the primary analytic sample (N_Sources ≥ 8; *n* = 211) with a broader sample (N_Sources ≥ 7; *n* = 251) evaluated the robustness of the inclusion criterion. Although the N_Sources ≥ 8 inclusion criterion constituted sample selection prior to analysis, REML and FIML estimation within the retained analytic sample used all available observations at each measurement occasion rather than applying additional listwise deletion.

Before examining longitudinal trajectories, measurement invariance of the Engagement Scale and MSLQ-M was evaluated using a sequence of confirmatory factor models estimated with WLSMV in Mplus Version 8.3 (configural, metric, and scalar). Invariance was evaluated using changes in CFI and RMSEA, with |ΔCFI| < 0.010 and |ΔRMSEA| < 0.015 indicating acceptable invariance (Chen, 2007; Cheung and Rensvold, 2002). For the MSLQ-M, due to model complexity, invariance was tested separately for each of the six subscales (Newsom, 2023).

Separate linear mixed models (LMM) were fitted for each engagement and SRL subscale with Timepoint (W1, W6, W11, and W15) specified as a fixed effect and a Compound Symmetry covariance structure. Significant omnibus effects were followed by Bonferroni-adjusted pairwise comparisons. To examine changes between consecutive instructional phases, Bonferroni-corrected paired-samples *t*-tests were additionally conducted. Effect sizes were reported using partial eta-squared (η^2^p) for the mixed models and Cohen's *d* for paired comparisons. The Compound Symmetry covariance structure was selected over unstructured and AR(1) alternatives based on BIC comparisons. Denominator degrees of freedom were estimated using the Satterthwaite approximation.

Longitudinal directional relationships among agentic engagement, metacognitive self-regulation, and academic achievement were examined using an observed-variable cross-lagged panel model (CLPM). The CLPM was specified as a directional rather than fully reciprocal model. Reverse cross-lagged paths from metacognitive self-regulation or academic achievement to agentic engagement were not estimated because the hypothesized theoretical model positioned agentic engagement as the antecedent of subsequent changes in self-regulated learning and academic achievement. Baseline covariances among the three constructs at Week 1 and theoretically justified within-wave residual covariances at Week 6 were freely estimated. Indirect effects were evaluated using bias-corrected bootstrap confidence intervals based on 1,000 bootstrap samples. As a sensitivity analysis, a Random Intercept Cross-Lagged Panel Model (RI-CLPM; Hamaker et al., 2015) was also estimated to separate stable between-person differences from within-person fluctuations. Because the achievement random intercept produced a non-positive definite PSI matrix, indicating model instability with the three-wave design, the RI-CLPM was interpreted as a robustness analysis rather than replacing the primary CLPM.

Descriptive statistics are reported as means (M) and standard deviations (SD). Model fit was assessed using the Comparative Fit Index (CFI) and Tucker–Lewis Index (TLI), with values of 0.95 or above indicating good fit and 0.90–0.95 acceptable fit; the Root Mean Square Error of Approximation (RMSEA), with values of 0.06 or below indicating good fit and values up to 0.08 indicating reasonable fit; and the Standardized Root Mean Square Residual (SRMR), with values of 0.08 or below indicating acceptable fit (Hu and Bentler, 1999). Competing models were compared using AIC and BIC, with lower values indicating better fit after accounting for model complexity. Effect sizes were interpreted using Cohen's (1988) guidelines, where partial η^2^ values of 0.01, 0.06, and 0.14 and Cohen's *d* values of 0.20, 0.50, and 0.80 represent small, medium, and large effects, respectively. Statistical significance was set at *p* < 0.05 (two-tailed). Internal consistency was evaluated using Cronbach's α, with values of 0.70 or above considered acceptable (Nunnally and Bernstein, 1994; Taber, 2018). Partial eta-squared values for the linear mixed models were derived from the reported F statistics and associated numerator and denominator degrees of freedom using η^2^p = (F × df1)/(F × df1 + df_2_).

## 4 Results

### 4.1 Preliminary analysis

Descriptive statistics and internal consistency coefficients for all engagement and SRL subscales across the four measurement timepoints are presented in Table 3. The primary longitudinal analytic sample comprised 211 students who met the inclusion criterion of providing data from at least 8 of the 10 possible data sources (N < sub>Sources < /sub> ≥ 8). After removing duplicate survey submissions, the numbers of unique respondents at each wave were W1 = 264, W6 = 259, W11 = 237, and W15 = 215. Baseline academic achievement on the 15-item knowledge test averaged 9.47 (SD = 2.82), increasing to 10.40 (SD = 2.69) following the standard flipped phase (W6) and 11.29 (SD = 2.70) at the end of the AI-enhanced phase (W15). Achievement scores also increased significantly between Week 6 and Week 15 (M = 10.46, SD = 2.70 vs. M = 11.31, SD = 2.67; *t* (212) = −4.76, *p* < 0.001, dz = 0.33), based on 213 participants with data at both timepoints, indicating continued knowledge acquisition during the AI-enhanced phase. AI learning log adherence was 90.3%, 84.5%, and 79.8% across Phases 1–3, respectively. Despite declining log submission rates, active generative AI use among submitters increased from 95.3% in Phase 1 to 98.5% in Phase 3.

### 4.2 Engagement trajectories across the standard to AI-enhanced transition (RQ1)

**Figure 1:**
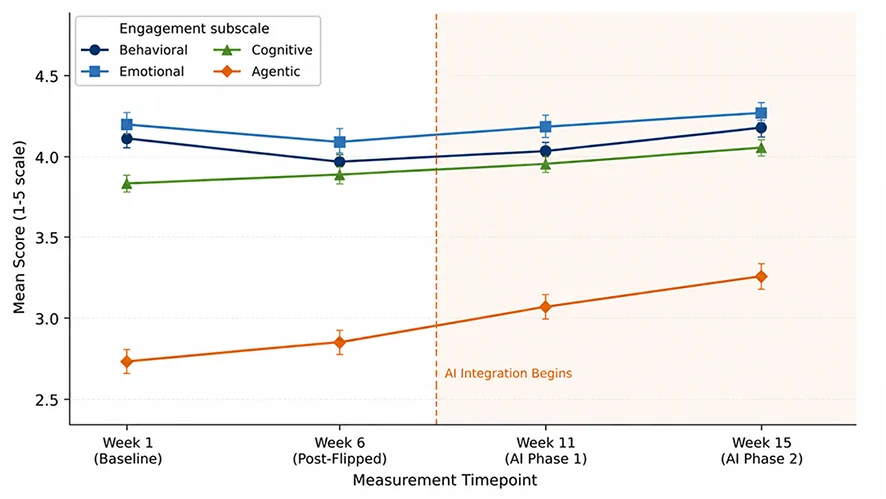
Developmental trajectories of the four student engagement dimensions across four measurement timepoints. Error bars represent ±1 SE. The shaded region indicates the AI-enhanced flipped phase (Weeks 7–15). BEH, Behavioral Engagement; EMO, Emotional Engagement; COG, Cognitive Engagement; AGEN, Agentic Engagement.

**Figure 2:**
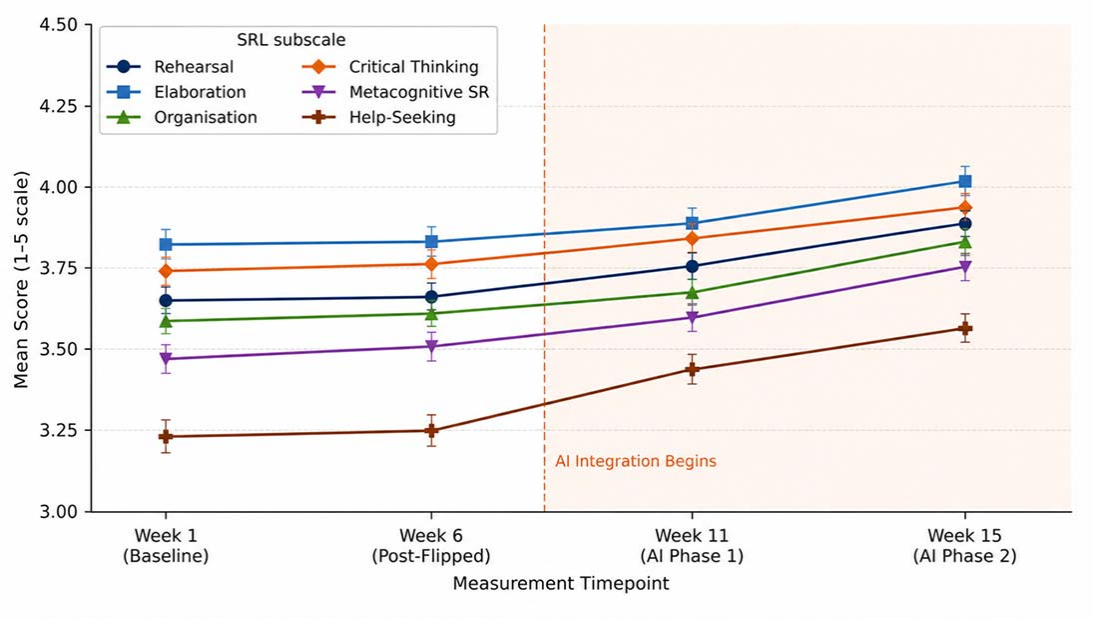
Developmental trajectories of the six self-regulated learning strategy subscales across four measurement timepoints. Error bars represent ±1 SE. The shaded region indicates the AI-enhanced flipped phase (Weeks 7–15). REH, Rehearsal; ELA, Elaboration; ORG, Organization; CT, Critical Thinking; METASR, Metacognitive Self-Regulation; HS, Help-Seeking.

To address RQ1, 10 linear mixed models (REML, Compound Symmetry covariance structure) were estimated to examine trajectories across W1, W6, W11, and W15 for the four engagement and six SRL subscales (Figures 1, 2). Academic achievement was analyzed separately because it was not assessed at W11. Phase-specific mean differences are presented in Table 4, and linear mixed model results are reported in Table 5. All 10 omnibus tests were statistically significant (all *p* ≤ 0.004), indicating significant longitudinal changes across the study period. Effect sizes ranged from small (η^2^p = 0.021 for Emotional Engagement) to medium (η^2^p = 0.111 for Metacognitive Self-Regulation), with Agentic Engagement (η^2^p = 0.103) showing the largest effect among the engagement dimensions.

**Table 4:** Phase-specific changes in engagement and SRL across standard flipped and AI-enhanced phases.

Subscale	W1–W6 (standard flipped)	W6–W11 (mid AI-enhanced phase)	W11–W15 (end AI-enhanced phase)	W1–W15 (overall)
Engagement				
Behavioral engagement	**Δ** **=** –0.15^***^ (*d* = −0.26)	Δ = +0.05 (*d* = +0.08)	**Δ** **=** +0.17^***^ (*d* = +0.28)	Δ = +0.08 (*d* = +0.12)
Emotional engagement	Δ = −0.10 (*d* = −0.13)	**Δ** **=** +0.10^*^ (*d* = +0.15)	Δ = +0.06 (*d* = +0.10)	Δ = +0.08 (*d* = +0.12)
Cognitive engagement	Δ = +0.04 (*d* = +0.08)	Δ = +0.06 (*d* = +0.10)	**Δ** **=** +0.12^**^ (*d* = +0.22)	**Δ** **=** +0.22^***^ (*d* = +0.33)
Agentic engagement	**Δ** **=** +0.13^*^ (*d* = +0.14)	**Δ** **=** +0.19^**^ (*d* = +0.19)	**Δ** **=** +0.22^**^ (*d* = +0.22)	**Δ** **=** +0.56^***^ (*d* = +0.53)
SRL strategies (MSLQ-M)				
Rehearsal	Δ = +0.01 (*d* = +0.01)	**Δ** **=** +0.15^**^ (*d* = +0.22)	**Δ** **=** +0.15^**^ (*d* = +0.21)	**Δ** **=** +0.30^***^ (*d* = +0.44)
Elaboration	Δ = +0.01 (*d* = +0.01)	Δ = +0.06 (*d* = +0.09)	**Δ** **=** +0.15^***^ (*d* = +0.24)	**Δ** **=** +0.21^***^ (*d* = +0.31)
Organization	Δ = +0.05 (*d* = +0.07)	Δ = +0.06 (*d* = +0.08)	**Δ** **=** +0.21^***^ (*d* = +0.28)	**Δ** **=** +0.32^***^ (*d* = +0.36)
Critical thinking	Δ = +0.03 (*d* = +0.04)	**Δ** **=** +0.11^*^ (*d* = +0.16)	Δ = +0.08 (*d* = +0.13)	**Δ** **=** +0.21^***^ (*d* = +0.29)
Metacognitive self-regulation	Δ = +0.06 (*d* = +0.10)	**Δ** **=** +0.11^**^ (*d* = +0.18)	**Δ** **=** +0.19^***^ (*d* = +0.31)	**Δ** **=** +0.35^***^ (*d* = +0.54)
Help-seeking	Δ = +0.01 (*d* = +0.01)	**Δ** **=** +0.17^**^ (*d* = +0.20)	**Δ** **=** +0.19^**^ (*d* = +0.21)	**Δ** **=** +0.36^***^ (*d* = +0.35)
**Achievement (ACHIE)**	**Δ** **=** +0.93^***^ (*d* = +0.34)	—	—	**Δ** **=** +1.82^***^ (*d* = +0.54)

Values represent mean differences (Δ) and Cohen's *d* for adjacent measurement occasions and the overall W1–W15 comparison. Paired-samples *t* tests were conducted using the primary longitudinal analytic sample (*N* = 211). W1–W6 represents the standard flipped phase. The W6–W11 comparison reflects changes from the end of the standard flipped phase to the midpoint of the AI-enhanced phase, encompassing the Guided and early Semi-Guided stages. The W11–W15 comparison reflects changes from the midpoint to the end of the AI-enhanced phase, encompassing the later Semi-Guided and Self-Directed stages. Achievement was assessed only at W1, W6, and W15; therefore, adjacent comparisons during the AI-enhanced phase are unavailable. Statistical significance is based on Bonferroni-adjusted paired-samples *t* tests. ^*^p < 0.05. ^**^ *p* < 0.01. ^***^ *p* < 0.001. Bold values indicate statistically significant results (*p* < 0.05).

**Table 5:** Linear mixed model results for engagement and SRL trajectories across four timepoints (*N* = 211).

Subscale	*F*	*p*	η^2^p	Size	Significant Bonferroni pairs
Engagement					
Behavioral engagement	10.84	< 0.001	0.050	small	W1 > W6 (*p* = 0.003); W6 < W15 (*p* < 0.001); W11 < W15 (*p* < 0.001)
Emotional engagement	4.52	0.004	0.021	small	W6 < W15 (*p* = 0.002)
Cognitive engagement	9.27	< 0.001	0.043	small	W1 < W15 (*p* < 0.001); W6 < W15 (*p* = 0.001)
Agentic engagement	23.69	< 0.001	0.103	medium	W1 < W11 (*p* < 0.001); W1 < W15 (*p* < 0.001); W6 < W11 (*p* = 0.025); W6 < W15 (*p* < 0.001); W11 < W15 (*p* = 0.010)
SRL strategies (MSLQ-M)					
Rehearsal	17.49	< 0.001	0.078	medium	W1 < W11 (*p* = 0.006); W1 < W15 (*p* < 0.001); W6 < W11 (*p* = 0.010); W6 < W15 (*p* < 0.001); W11 < W15 (*p* = 0.020)
Elaboration	9.09	< 0.001	0.042	small	W1 < W15 (*p* < 0.001); W6 < W15 (*p* < 0.001); W11 < W15 (*p* = 0.011)
Organization	12.23	< 0.001	0.056	small	W1 < W15 (*p* < 0.001); W6 < W15 (*p* < 0.001); W11 < W15 (*p* = 0.001)
Critical thinking	8.44	< 0.001	0.039	small	W1 < W11 (*p* = 0.025); W1 < W15 (*p* < 0.001); W6 < W15 (*p* = 0.001)
Metacognitive self-regulation	25.97	< 0.001	0.111	medium	W1 < W11 (*p* = 0.001); W1 < W15 (*p* < 0.001); W6 < W15 (*p* < 0.001); W11 < W15 (*p* < 0.001)
Help-seeking	13.23	< 0.001	0.060	medium	W1 < W11 (*p* = 0.029); W1 < W15 (*p* < 0.001); W6 < W11 (*p* = 0.047); W6 < W15 (*p* < 0.001)

Linear mixed models were estimated using restricted maximum likelihood (REML) with a compound symmetry covariance structure. Timepoint (W1, W6, W11, W15) was specified as the fixed effect. Partial eta-squared (η^2^p) is reported as the measure of effect size and interpreted using Cohen's (1988) benchmarks: small = 0.01, medium = 0.06, and large = 0.14. Bonferroni-adjusted pairwise comparisons are reported only for statistically significant contrasts. W1 = Week 1 (baseline); W6 = Week 6 (post-standard flipped phase); W11 = Week 11 (mid-AI-enhanced phase); W15 = Week 15 (post-AI-enhanced phase).

*Behavioral engagement:* behavioral engagement showed a significant trajectory across the four measurement occasions, *F* (3, 618.62) = 10.843, *p* < 0.001, η^2^p = 0.050. Bonferroni-adjusted pairwise comparisons showed a significant decrease from W1 to W6 (*p* = 0.003). Scores remained stable at W11 before increasing significantly between W6 and W15 (*p* < 0.001) and between W11 and W15 (*p* < 0.001). The comparison between W1 and W15 was not statistically significant (*p* = 0.487), indicating that behavioral engagement returned to a level comparable with baseline by the end of the study.

*Emotional engagement:* emotional engagement also showed a statistically significant overall trajectory, *F* (3, 619.59) = 4.516, *p* = 0.004, η^2^p = 0.021 (small effect). Bonferroni-adjusted pairwise comparisons indicated that only one comparison reached statistical significance: scores at W15 were significantly higher than those at W6 (*p* = 0.002). No other pairwise comparisons were significant. Emotional engagement was highest at W15 (*M* = 4.27), compared with W6 (*M* = 4.10) and baseline (W1: *M* = 4.20).

*Cognitive engagement:* cognitive engagement increased significantly across the study period, *F* (3, 621.93) = 9.268, *p* < 0.001, η^2^p = 0.043 (small effect). Bonferroni-adjusted pairwise comparisons showed that scores at W15 were significantly higher than those at W1 (*p* < 0.001) and W6 (*p* = 0.001). No other adjacent-wave comparisons reached statistical significance. Cognitive engagement showed a modest increase across the study period, with significantly higher scores at W15 than at W1 and W6.

*Agentic engagement:* agentic engagement demonstrated the strongest trajectory among the engagement dimensions, *F* (3, 621.33) = 23.688, *p* < 0.001, η^2^p = 0.103 (medium effect). Five of the six Bonferroni-adjusted pairwise comparisons were statistically significant. Beginning with the lowest baseline mean among the engagement dimensions (W1: *M* = 2.75), agentic engagement increased significantly between W1 and W11 (*p* < 0.001), W1 and W15 (*p* < 0.001), W6 and W11 (*p* = 0.025), W6 and W15 (*p* < 0.001), and W11 and W15 (*p* = 0.010). By W15, the mean score had increased to 3.29, representing an overall gain of 0.56 points across the study period.

Overall, the findings indicate that the four engagement dimensions followed distinct developmental trajectories across the transition from standard to AI-enhanced flipped instruction, with agentic engagement showing the largest and most sustained increase over time.

### 4.3 SRL strategy use across the standard to AI-enhanced transition (RQ2)

To address RQ2, a consistent longitudinal pattern emerged across all six SRL subscales (Figure 2). No significant Bonferroni-adjusted changes occurred during the standard flipped phase (W1–W6), whereas all six subscales increased significantly during the AI-enhanced phase (W6–W15), although the timing and magnitude of change differed across strategies. Metacognitive Self-Regulation (η^2^p = 0.111), Rehearsal (η^2^p = 0.078), and Help-Seeking (η^2^p = 0.060) demonstrated the largest effect sizes. Help-Seeking showed the earliest significant improvement, becoming higher than baseline by W11 (*p* = 0.029), whereas Elaboration and Organization exhibited their largest gains during the final instructional phase (W11–W15). Metacognitive Self-Regulation showed the greatest overall increase (ΔM = +0.35).

*Rehearsal:* rehearsal strategy use showed a significant trajectory across the four measurement occasions, *F* (3, 622.24) = 17.490, *p* < 0.001, η^2^p = 0.078 (medium effect). Scores remained unchanged between W1 (*M* = 3.65) and W6 (*M* = 3.65; p = 1.000). Significant increases were observed between W6 and W11 (p = 0.010) and between W11 and W15 (*p* = 0.020). Overall, rehearsal increased significantly from baseline to W15 after remaining unchanged during the standard flipped phase.

*Elaboration:* elaboration strategy use increased significantly across the study period, *F* (3, 622.17) = 9.091, *p* < 0.001, η^2^p = 0.042 (small effect). No significant difference was observed between W1 and W6 (*p* = 1.000) or between W6 and W11 (*p* = 0.778). Significant increases occurred between W11 and W15 (*p* = 0.011), with scores at W15 significantly higher than those at both W1 (*p* < 0.001) and W6 (*p* < 0.001). Mean elaboration increased from 3.79 at baseline to 4.01 at W15.

*Organization:* organization strategy use showed a significant overall trajectory, F (3, 621.25) = 12.232, *p* < 0.001, η^2^p = 0.056 (small effect). Like elaboration, scores remained stable across W1, W6, and W11, with no significant adjacent-wave comparisons until the final phase. The significant leap from W11 to W15 (*p* = 0.001) drove most of the overall effect, with W15 (*M* = 3.89) significantly higher than W1 (*p* < 0.001), W6 (*p* < 0.001), and W11 (*p* = 0.001).

*Critical thinking:* critical thinking strategy use increased significantly across the study period, *F* (3, 622.39) = 8.440, *p* < 0.001, η^2^p = 0.039 (small effect). Scores did not differ significantly between W1 and W6 (*p* = 1.000). W11 scores were significantly higher than W1 (*p* = 0.025), and W15 scores were significantly higher than both W1 (*p* < 0.001) and W6 (*p* = 0.001). The comparison between W11 and W15 was not statistically significant (*p* = 0.710), indicating that scores remained stable during the final phase of the study.

*Metacognitive self-regulation:* metacognitive self-regulation demonstrated the strongest trajectory among the SRL subscales, *F* (3, 621.66) = 25.967, *p* < 0.001, η^2^p = 0.111 (medium effect). Scores remained stable between W1 and W6 (*p* = 1.000), increased significantly by W11 relative to baseline (*p* = 0.001), and increased further between W11 and W15 (*p* < 0.001). The cumulative increase from W1 to W15 was 0.35 points, representing the largest gain among the SRL dimensions.

*Help-seeking:* help-seeking strategy use increased significantly across the study period, *F* (3, 621.45) = 13.226, *p* < 0.001, η^2^p = 0.060 (medium effect). Scores remained stable during the standard flipped phase (W1 vs. W6, *p* = 1.000). Significant increases were observed at W11 relative to both W1 (*p* = 0.029) and W6 (*p* = 0.047), with further increases evident at W15 relative to W1 (*p* < 0.001) and W6 (*p* < 0.001). The comparison between W11 and W15 approached statistical significance (*p* = 0.052).

Overall, these findings answer RQ2 by showing that SRL strategies remained stable during the standard flipped phase before increasing during the AI-enhanced phase, with metacognitive self-regulation demonstrating the largest overall improvement.

### 4.4 Largest changes across instructional transitions (RQ3)

Addressing RQ3, distinct developmental patterns emerged across the engagement and SRL domains (Table 5; Figures 1, 2). Behavioral engagement followed a U-shaped trajectory, declining during the standard flipped phase before recovering during the AI-enhanced phase. Emotional engagement showed relatively modest change across the study period. Cognitive engagement and agentic engagement increased over time, with agentic engagement demonstrating the largest cumulative improvement among the engagement dimensions (ΔM = +0.56, *d* = 0.53).

Across the SRL strategies, improvements emerged only during the AI-enhanced phase, with Metacognitive Self-Regulation (η^2^p = 0.111), Rehearsal (η^2^p = 0.078), and Help-Seeking (η^2^p = 0.060) showing the largest overall effects. The greatest adjacent-wave improvements occurred during the final instructional period (W11–W15), particularly for Organization, Elaboration, and Metacognitive Self-Regulation. Academic achievement also increased significantly across the study, improving by 1.82 points between W1 and W15 (*d* = 0.54).

Overall, these findings answer RQ3 by indicating that the largest engagement gains occurred for agentic engagement across the study, whereas the largest SRL gains were observed during the W11–W15 interval, which spans the Semi-Guided and Self-Directed stages of AI integration.

### 4.5 Cross-lagged relationships among agentic engagement, metacognitive self-regulation, and academic achievement (RQ4)

**Figure 3:**
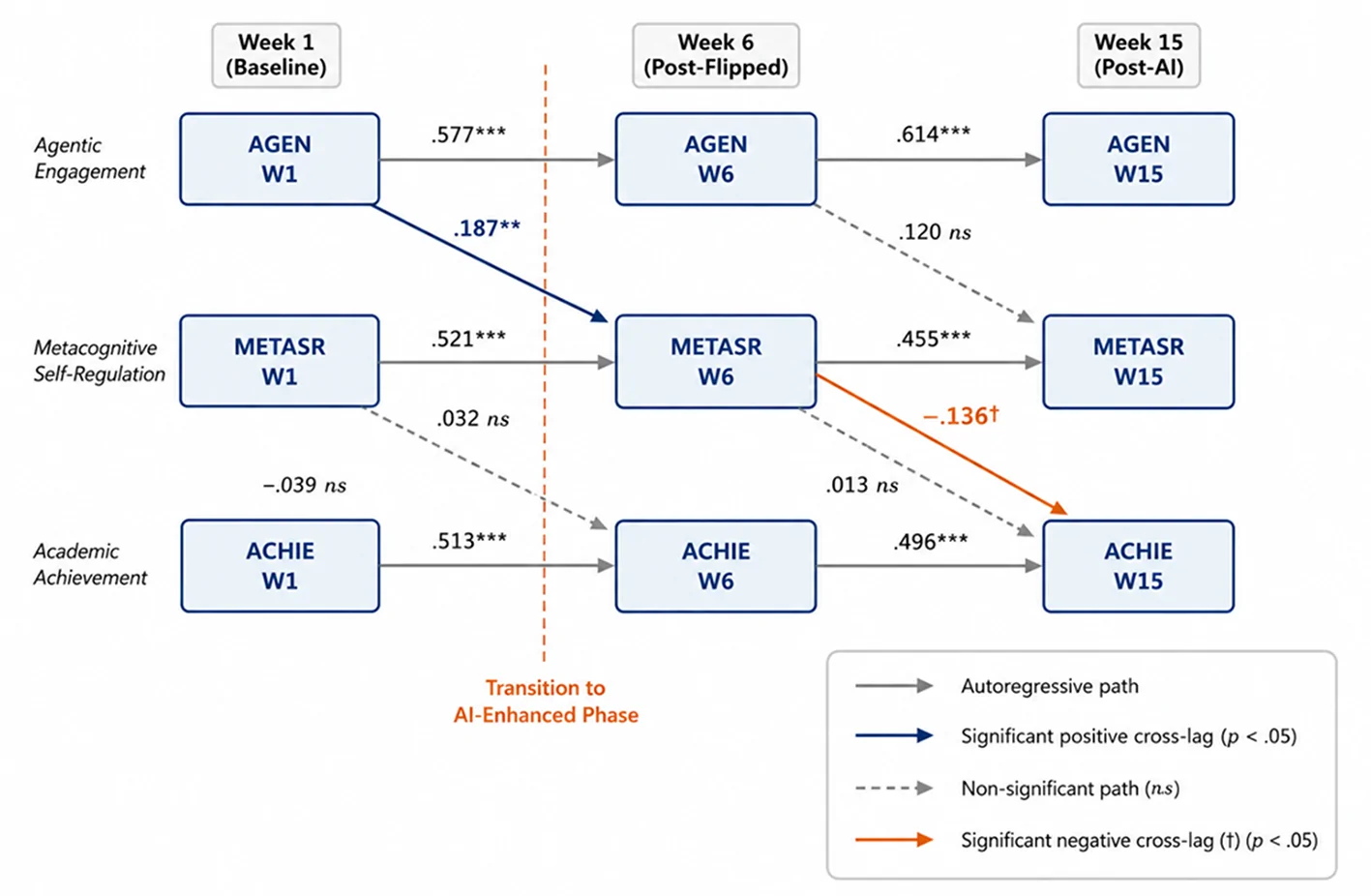
Cross-lagged panel model showing standardized path coefficients among Agentic Engagement, Metacognitive Self-Regulation, and Academic Achievement across Weeks 1, 6, and 15. Gray solid arrows represent autoregressive paths, blue solid arrows represent statistically significant positive cross-lagged paths, gray dashed arrows represent non-significant paths, and the orange solid arrow represents a statistically significant negative cross-lagged path. Standardized path coefficients are reported in Table 6.

To address RQ4, longitudinal directional relationships among agentic engagement, metacognitive self-regulation, and academic achievement were examined using the prespecified directional cross-lagged panel model (CLPM). The model was estimated using the three primary measurement occasions (Weeks 1, 6, and 15); consequently, the intermediate Week 11 assessment was not included in the analysis. Figure 3 presents the final model, and Table 6 reports the standardized path coefficients and bias-corrected bootstrap confidence intervals. Model fit is reported first, followed by the autoregressive and cross-lagged relationships, robustness analyses, and a summary of the findings.

**Table 6:** Standardized cross-lagged panel model path coefficients (*N* = 211).

Path	β (std.)	SE	*p*	95% BC CI
Autoregressive paths				
AGEN W1 → AGEN W6	0.577	0.049	< 0.001	(0.469, 0.659)
AGEN W6 → AGEN W15	0.614	0.050	< 0.001	(0.503, 0.705)
METASR W1 → METASR W6	0.521	0.059	< 0.001	(0.403, 0.631)
METASR W6 → METASR W15	0.455	0.065	< 0.001	(0.330, 0.583)
ACHIE W1 → ACHIE W6	0.513	0.055	< 0.001	(0.403, 0.615)
ACHIE W6 → ACHIE W15	0.496	0.065	< 0.001	(0.363, 0.616)
Cross-lagged paths				
AGEN W1 → METASR W6	0.187	0.066	0.005	(0.064, 0.315)
AGEN W6 → METASR W15	0.120	0.080	0.136	(−0.042, 0.273)
METASR W1 → ACHIE W6	0.032	0.068	0.638	(−0.103, 0.170)
METASR W6 → ACHIE W15	0.013	0.075	0.861	(−0.127, 0.169)
Direct paths				
AGEN W1 → ACHIE W6	−0.039	0.074	0.598	(−0.179, 0.103)
AGEN W6 → ACHIE W15	−0.136	0.067	0.042	(−0.266, −0.008)
Indirect effect				
AGEN W1 → METASR W6 → ACHIE W15	0.002	0.015	0.872	(−0.028, 0.035)

The model was estimated in Mplus 8.3 using maximum likelihood estimation with full information maximum likelihood (FIML) to handle missing data. Standardized (STDYX) path coefficients, standard errors, *p* values, and 95% bias-corrected bootstrap confidence intervals are reported. β, standardized path coefficient; BC CI, bias-corrected confidence interval; AGEN, Agentic Engagement; METASR, Metacognitive Self-Regulation; ACHIE, Academic Achievement.

#### 4.5.1 Model fit

Two nested cross-lagged panel models were estimated in Mplus Version 8.3 using maximum likelihood estimation with full information maximum likelihood (FIML) to accommodate the seven participants with missing Week 15 data. Model 1 included only autoregressive stability paths, whereas Model 2 additionally included the hypothesized cross-lagged and direct paths specified for RQ4. Relative to Model 1, Model 2 showed improved CFI (0.896–0.922; ΔCFI = +0.026) and SRMR (0.109–0.079), whereas RMSEA increased slightly (0.107–0.118) and TLI remained largely unchanged (0.850 vs. 0.848). Information criteria provided mixed evidence, with AIC favoring Model 2 (ΔAIC = −5.87) and BIC favoring the more parsimonious Model 1 (ΔBIC = +14.25).

Although TLI (0.848) and RMSEA (0.118) did not meet the conventional fit criteria specified in the Data Analysis section, observed-variable CLPMs often exhibit less favorable global fit than latent-variable models, particularly when degrees of freedom are limited (Hamaker et al., 2015; Kenny et al., 2015). Accordingly, the CLPM findings are interpreted cautiously and in conjunction with the longitudinal trajectory analyses.

#### 4.5.2 Autoregressive paths

All six autoregressive paths were statistically significant (all *p* < 0.001), indicating moderate-to-strong temporal stability for all three constructs (Table 6). Agentic engagement demonstrated increasing stability from Week 1–Week 6 (β = 0.577) to Week 6–Week 15 (β = 0.614). Metacognitive self-regulation also showed moderate stability across both intervals (β = 0.521 and β = 0.455), as did academic achievement (β = 0.513 and β = 0.496). These findings indicate substantial within-construct stability across the study period.

#### 4.5.3 Cross-lagged paths and RQ4

RQ4 examined whether metacognitive self-regulation mediated the longitudinal relationship between agentic engagement and academic achievement. Higher agentic engagement at Week 1 significantly predicted higher metacognitive self-regulation at Week 6 [β = 0.187, SE = 0.066, *p* = 0.005, 95% CI (.064, 0.315)]. However, metacognitive self-regulation at Week 6 did not significantly predict academic achievement at Week 15 [β = 0.013, SE = 0.075, *p* = 0.861, 95% CI (−0.127, 0.169)]. Consequently, the indirect effect of agentic engagement on academic achievement through metacognitive self-regulation was small and non-significant [β = 0.002, SE = 0.015, *p* = 0.872, 95% BC CI (−0.028, 0.035)], providing no support for the hypothesized mediation.

One additional direct path reached statistical significance. Higher agentic engagement at Week 6 negatively predicted academic achievement at Week 15 [β = −0.136, SE = 0.067, *p* = 0.042, 95% CI (−0.266, −0.008)]. This unexpected finding is considered further in the Discussion.

#### 4.5.4 Robustness analysis

As a robustness analysis, a Random Intercept Cross-Lagged Panel Model (RI-CLPM; Hamaker et al., 2015) was estimated. Model fit improved substantially relative to the standard CLPM (CFI = 0.981, TLI = 0.947, RMSEA = 0.070, SRMR = 0.036). The random intercepts for agentic engagement and metacognitive self-regulation were strongly correlated (*r* = 0.732, *p* < 0.001), indicating stable between-person covariation. However, none of the within-person cross-lagged paths reached statistical significance (all *p* > 0.05), including the path from Week 1 agentic engagement to Week 6 metacognitive self-regulation (β = 0.069, *p* = 0.479), and the indirect effect remained non-significant [β = −0.007, 95% BC CI (−0.075, 0.015)]. The random intercept for academic achievement could not be estimated reliably because of a non-positive definite PSI matrix, consistent with overparameterization given the three-wave design. Accordingly, the RI-CLPM findings are interpreted as a robustness analysis rather than replacing the primary CLPM results.

#### 4.5.5 Summary of RQ4 findings

Overall, the cross-lagged analyses provided partial support for RQ4. Agentic engagement positively predicted subsequent metacognitive self-regulation, indicating a prospective longitudinal association between proactive classroom participation and later self-regulatory strategy use. However, metacognitive self-regulation did not predict subsequent academic achievement, and the hypothesized indirect effect was not supported. These findings suggest that although agentic engagement may facilitate later development of metacognitive self-regulation, this pathway did not translate into improved academic achievement within the timeframe of the present study.

## 5 Discussion

### 5.1 The dormant-then-activated SRL pattern (RQ2 and RQ3)

The most theoretically significant finding, addressing RQ2, was the consistent pattern observed across all six SRL subscales: no significant growth during the standard flipped phase (all Bonferroni-adjusted W1–W6 comparisons, *p* = 1.000), followed by significant increases during the AI-enhanced phase. Because the same students, instructor, and course were observed across both instructional phases, this pattern is consistent with the progressive introduction of AI scaffolding as an important contextual factor associated with SRL development. However, the absence of a parallel control group means that this interpretation should be viewed cautiously, as other time-related influences cannot be excluded.

One possible interpretation is that the standard flipped phase created opportunities for active learning without necessarily prompting students to regulate their learning strategically. In contrast, the AI-enhanced phase required students to formulate prompts, evaluate AI-generated responses, and iteratively refine their queries, behaviors that closely correspond to metacognitive monitoring and strategic regulation. This interpretation is consistent with Chi and Wylie's (2014) ICAP framework, which proposes that constructive and interactive learning activities produce deeper learning than active engagement alone. It also aligns with recent meta-analytic evidence demonstrating that ChatGPT-supported learning produces moderate-to-large improvements in self-regulation and cognitive engagement when students interact dialogically with AI rather than passively consume AI-generated content (Heung and Chiu, 2025). The AI learning logs collected throughout the intervention provide contextual support for this interpretation, indicating consistently high and increasing engagement with progressively scaffolded GenAI activities across the three instructional phases. Similarly, the meta-analysis by Xu et al. (2026) reported stronger effects of AI-supported learning during the performance phase of self-regulated learning than during the forethought phase, a pattern that closely resembles the dormant-then-activated trajectory observed in the present study. From Zimmerman's (2000) cyclical model of self-regulated learning, progressively scaffolded AI interactions may have strengthened learners' monitoring and control processes during the performance phase, thereby creating greater opportunities for strategic regulation than were available during the standard flipped phase.

A complementary explanation can be drawn from the perspective of co-regulation. Unlike the standard flipped phase, the AI-enhanced phase provided students with an immediately responsive conversational partner capable of prompting questioning, feedback, and iterative refinement. Such interactions have increasingly been conceptualized as forms of human–AI co-regulation that externalize aspects of metacognitive monitoring while leaving ultimate responsibility with the learner (Dahri et al., 2024; Järvelä et al., 2023). Importantly, these benefits are not automatic. Previous work has emphasized that AI supports self-regulation only when learners critically evaluate AI-generated responses rather than accepting them uncritically (Kasneci et al., 2023). Accordingly, instructors should design AI-supported learning activities that require students to justify, verify, and extend AI-generated responses instead of treating AI as an answer-generating tool.

With respect to RQ3, the largest SRL gains occurred during the final AI-supported instructional period, whereas earlier AI phases produced more modest improvements. This pattern suggests that progressively reducing instructional support may encourage students to assume increasing responsibility for regulating their own learning. Rather than producing immediate changes, AI-supported self-regulation appeared to strengthen as learners became more autonomous in their interactions with AI. Beyond the overall increase in SRL, the longitudinal trajectories suggest a developmental progression from relatively simple information acquisition strategies toward increasingly complex information transformation strategies. Rehearsal and help-seeking showed earlier improvements following AI integration, whereas elaboration, organization, and critical thinking exhibited their largest gains during the W11–W15 interval. Although this interpretation should be considered tentative, it is consistent with the idea that progressively scaffolded AI use may initially support information acquisition before fostering deeper knowledge transformation.

A complementary interpretation is that the standard flipped phase functioned not as a pedagogically ineffective precursor but as a necessary period of disciplinary knowledge building. 6 weeks of second-language acquisition content, discussion, and collaborative problem-solving may have established the conceptual foundation that later enabled students to engage more strategically with AI-supported learning. Under this interpretation, strategic learning behaviors did not remain dormant because flipped instruction failed, but because students were still acquiring the domain knowledge needed to benefit fully from AI-supported inquiry. Future research should directly examine whether prior content mastery moderates the effectiveness of AI-enhanced flipped learning for developing self-regulated learning. Taken together, these findings are consistent with a two-stage developmental model of AI-enhanced self-regulated learning. In this model, the standard flipped phase may establish conditions conducive to strategic regulation, whereas progressively scaffolded AI interactions provide opportunities for regulatory processes to develop. Given the within-subjects design and the absence of a parallel control group, this developmental model should be considered a hypothesis for future experimental and counterbalanced longitudinal research rather than a causal explanation.

### 5.2 Engagement trajectories and the role of agentic engagement (RQ1)

Addressing RQ1, agentic engagement demonstrated the strongest and most consistent developmental trajectory, showing significant growth across every instructional transition and the largest overall effect among the engagement dimensions (η^2^p = 0.103). This finding is consistent with the conceptualization of agentic engagement as students‘ proactive contribution to shaping their own learning environment (Reeve's, 2013; Reeve and Tseng's, 2011). It also aligns with longitudinal evidence showing that students who display greater agentic engagement subsequently perceive higher levels of autonomy-supportive teaching, suggesting a reciprocal process in which students actively influence their own learning context (Jang et al., 2024; Matos et al., 2018). In the present study, progressively reducing instructional scaffolding while increasing students' responsibility for directing AI interactions may have created increasingly favorable conditions for agentic engagement to develop. This interpretation is further supported by recent reviews identifying opportunities for choice, self-initiation, and inquiry as central conditions for fostering agentic engagement (Utami and Kurniawati, 2024).

Taken together, these findings reinforce growing theoretical arguments that agentic engagement may represent an important mechanism through which AI-supported learning environments promote deeper self-regulation. Rather than simply participating in learning activities, students increasingly initiated, directed, and evaluated their interactions with AI, behaviors that closely reflect the defining characteristics of agentic engagement.

Behavioral and emotional engagement followed similar recovery trajectories. Behavioral engagement initially declined during the standard flipped phase before returning to baseline levels during the AI-enhanced phase, whereas emotional engagement showed a smaller decline followed by gradual improvement, reaching its highest level at the end of the semester. One possible explanation for this initial decline is that students initially experienced increased cognitive demands while adapting to the unfamiliar flipped classroom format, consistent with Cognitive Load Theory (Sweller, 2023). This interpretation also aligns with previous reviews identifying inadequate preparation for out-of-class learning as one of the most common implementation challenges in flipped classrooms (Akçayir and Akçayir, 2018). As students became more familiar with the instructional approach and increasingly integrated AI into their learning, these initial adjustment costs may have diminished.

Cognitive engagement also increased across the study, with the largest gains occurring during the later AI-enhanced phase. This pattern is consistent with the ICAP framework (Chi and Wylie's, 2014), which proposes that deeper cognitive engagement emerges when learners move beyond active participation toward constructive and interactive knowledge building. In the present study, progressively scaffolded AI activities required students not only to retrieve information but also to evaluate, refine, and integrate AI-generated responses into their own understanding. These increasingly demanding interactions may help explain why cognitive engagement developed more gradually than agentic engagement.

Overall, the engagement findings suggest that the four dimensions responded differently across the intervention. Behavioral and emotional engagement appeared to reflect students' adaptation to a new instructional format, whereas cognitive engagement, and particularly agentic engagement, was more closely associated with progressively increasing opportunities for learner autonomy and self-directed interaction with AI. This interpretation is also consistent with the longitudinal analyses, which showed that higher agentic engagement at Week 1 predicted subsequent metacognitive self-regulation at Week 6, although the hypothesized indirect effect on academic achievement was not supported.

### 5.3 Cross-lagged panel model findings (RQ4)

#### 5.3.1 Interpreting the achievement finding

The cross-lagged panel model provided partial support for RQ4. Agentic engagement at Week 1 significantly predicted metacognitive self-regulation at Week 6 (β = 0.187, *p* = 0.005), extending previous evidence linking agentic engagement and self-regulation from predominantly cross-sectional research (Reeve and Tseng's, 2011) to a longitudinal context. However, metacognitive self-regulation did not significantly predict subsequent academic achievement, and a small negative direct association was observed between agentic engagement at Week 6 and academic achievement at Week 15 (β = −0.136, *p* = 0.042). A robustness analysis using a Random Intercept Cross-Lagged Panel Model did not replicate the within-person cross-lagged effects. Because the RI-CLPM was estimated from only three waves and exhibited estimation problems for academic achievement, these robustness findings should be interpreted cautiously and primarily as indicating the need for replication using longer longitudinal designs.

One possible explanation concerns the nature of the achievement measure. Academic achievement was assessed using a multiple-choice test designed to measure declarative knowledge of second-language acquisition concepts, whereas metacognitive self-regulation reflects learners' planning, monitoring, and evaluation of their own learning processes. These constructs represent different aspects of learning, and improvements in metacognitive regulation may not be immediately reflected in short-term factual knowledge tests (Panadero, 2017). Rather, improvements in metacognitive regulation may become more apparent in assessments requiring transfer, problem-solving, or application of knowledge over longer periods. This interpretation is consistent with the experimental study by Xu et al. (2025), who reported significant improvements in self-regulated learning following GenAI-supported instruction without corresponding improvements in academic achievement.

A second possibility is that the significant negative path from agentic engagement at W6 to academic achievement at W15 reflects a temporary redistribution of students' cognitive resources and learning efforts. During the middle stages of the intervention, students were encouraged to experiment with prompt construction, evaluate AI-generated responses, and develop effective AI-supported learning strategies. It is possible that greater investment in these exploratory learning activities temporarily displaced attention from preparing for a declarative knowledge assessment. Because this explanation cannot be tested directly with the present data, it should be regarded as speculative.

A third possibility is that unmeasured variables influenced the observed relationship. For example, differences in concurrent academic workload, motivation, or assessment priorities across students were not included in the model and may have affected achievement independently of engagement or self-regulation. Future studies should incorporate broader indicators of academic performance, including performance-based assessments and open-ended tasks, together with measures of students' overall academic workload, to better understand how AI-supported self-regulation translates into learning outcomes.

#### 5.3.2 Agentic engagement as a precursor to later self-regulation

Despite the absence of support for the full mediation model, the significant longitudinal path from agentic engagement at W1 to metacognitive self-regulation at W6 represents an important theoretical finding. Reeve and Tseng's (2011), building on Reeve's (2013) broader conceptualization of agentic engagement, described agentic engagement as students' proactive contribution to shaping their own learning through questioning, expressing preferences, and making suggestions. The present findings extend this perspective by showing that higher initial agentic engagement predicted subsequent metacognitive self-regulation over time.

This finding is consistent with Zimmerman's (2000) cyclical model of self-regulated learning, in which learners' proactive engagement during the forethought phase supports strategic monitoring and regulation of learning during the performance phase. Students who actively shaped their learning during the standard flipped phase subsequently reported stronger metacognitive regulation, suggesting that agentic engagement may function as an important precursor to later self-regulatory behavior.

The second cross-lagged path, from agentic engagement at Week 6 to metacognitive self-regulation at Week 15, was positive but did not reach statistical significance (β = 0.120, *p* = 0.136). Accordingly, the significant longitudinal association observed in the present study was limited to the first interval of the intervention. Importantly, this interval corresponded entirely to the standard flipped phase before AI-enhanced activities were introduced. Thus, the significant relationship between agentic engagement and later metacognitive self-regulation emerged within the conventional flipped learning context rather than during the AI-enhanced phase. Future studies with more frequent measurement occasions are needed to determine whether similar longitudinal associations emerge during later phases of AI-supported learning.

More broadly, the present findings extend previous longitudinal research demonstrating that agentic engagement predicts later improvements in students' learning environments and motivational functioning across diverse educational contexts (Jang et al., 2024; Matos et al., 2018). Together with the engagement and SRL trajectory analyses, the present findings support the proposition that agentic engagement may act as a precursor to later metacognitive self-regulation during flipped learning, a relationship that warrants further investigation in experimental and counterbalanced designs.

### 5.4 Theoretical and practical implications

The combined trajectory and cross-lagged findings provide preliminary support for the proposed two-stage developmental model of AI-enhanced self-regulated learning. During the standard flipped phase, agentic engagement predicted subsequent metacognitive self-regulation, yet observable growth in SRL strategies remained limited. During the AI-enhanced phase, all six SRL strategies increased significantly. Collectively, these findings suggest a developmental sequence in which the standard flipped classroom may have established motivational and behavioral conditions conducive to later strategic regulation, whereas progressively scaffolded AI interactions may have provided opportunities for these regulatory processes to develop. This pattern accords with the ICAP framework (Chi and Wylie's, 2014), which proposes that deeper learning emerges as learners progress from active to constructive and interactive engagement. It also aligns with Zimmerman's (2000) cyclical model of self-regulated learning, in which forethought processes support subsequent performance and self-reflection. These interpretations should be regarded as hypotheses generated by the present data rather than conclusions, pending experimental verification. Accordingly, the present study contributes to emerging theoretical perspectives on AI-supported learning by suggesting that agentic engagement may precede and facilitate the development of metacognitive self-regulation within progressively scaffolded learning environments.

From a practical perspective, the findings suggest that how AI is pedagogically integrated may be more important than whether AI is used. The progressive scaffolding approach, moving from guided to semi-guided and ultimately self-directed AI use, was associated with significant improvements across all SRL dimensions. Rather than providing unrestricted AI access from the outset, instructors may benefit from gradually transferring increasing responsibility for prompt design, evaluation of AI responses, and strategic decision-making to students as their confidence and regulatory skills develop.

The help-seeking finding warrants particular attention. AI tools may lower the threshold for help-seeking by allowing students to seek assistance without the hesitation or social judgment sometimes associated with asking questions in class, potentially leading to more proactive academic inquiry (Heung and Chiu, 2025; Ng et al., 2024). This may be particularly relevant in educational contexts where public help-seeking carries face-threatening social costs, including settings in Mongolia and other parts of Central Asia, where instructor authority and peer evaluation norms have been reported to discourage students from asking questions openly. The present findings suggest that AI-enhanced flipped classrooms may support meaningful SRL development (η^2^p = 0.060–0.111) within this context, providing encouraging evidence for institutions considering AI integration in educational settings where passive learning traditions remain common. However, because cultural beliefs and classroom norms were not measured directly, this interpretation should be regarded as tentative and warrants further investigation across diverse educational contexts.

At a broader pedagogical level, these findings suggest a shift in the educator's role from knowledge transmitter to learning facilitator who intentionally designs and scaffolds productive student and AI interactions. The effectiveness of this approach is also likely to depend on gradually reducing structured scaffolding, such as instructor-provided prompt templates, as students develop greater confidence and autonomy in directing their own AI-supported learning. Taken together, these findings suggest that the educational value of AI lies not simply in providing access to generative tools, but in intentionally designing learning environments that progressively cultivate learner autonomy, strategic engagement, and self-regulated learning.

### 5.5 Limitations and future directions

Several limitations should be acknowledged. First, the within-subjects design without a parallel control group prevents causal attribution. Although the longitudinal transition from standard to AI-enhanced instruction provides stronger evidence than cross-sectional designs, future randomized or quasi-experimental studies are needed to determine whether progressively scaffolded AI support contributes to SRL development.

Second, both instruments carry measurement limitations. The Critical Thinking subscale of the MSLQ-M underwent substantial cultural adaptation, shifting from idea generation toward ability application to reflect norms of teacher-directed learning; this subscale should therefore be interpreted as reflecting a contextually adapted conceptualization of critical thinking, which should be considered when generalizabi CT-specific findings. The convergent validity of four MSLQ-M subscales was limited, with AVE values below the recommended 0.50 threshold (Rehearsal, Elaboration, Organization, and Metacognitive Self-Regulation), suggesting these subscales should be interpreted with appropriate caution.

Academic achievement was assessed using the same 15-item multiple-choice test across three occasions; although item order was randomised, familiarity effects cannot be fully excluded, and two items showed negative discrimination coefficients and one showed a ceiling effect, limiting the sensitivity of the test to detect individual differences at the upper end of the ability distribution. Moreover, knowledge-based multiple-choice assessments may be less sensitive to process-level improvements in metacognitive regulation and strategic learning. Future studies should consider parallel test forms and complement knowledge tests with performance-based or authentic assessments that better capture higher-order learning outcomes.

Third, attrition analysis revealed that the analytic sample skewed toward students with higher baseline behavioral engagement, emotional engagement, and help-seeking relative to excluded participants, which may limit generalizability to less engaged student populations. Additionally, participants were drawn from a single university and the sample was predominantly female (88.6%), reflecting the enrolment profile of the participating programme. These characteristics may limit the generalizability of the findings to other institutional contexts or more gender-balanced student populations.

The directional longitudinal path model showed acceptable but not fully satisfactory fit: CFI = 0.922 exceeded the 0.90 threshold but TLI = 0.848 and RMSEA = 0.118 fell below the authors' own stated criteria of 0.95 and 0.06 respectively, indicating that fit was not fully satisfactory (Kenny et al., 2015). An RI-CLPM was estimated as a robustness check but produced an unstable random intercept for achievement (non-positive definite PSI matrix), likely reflecting model overparameterisation given the three-wave design. Future studies with additional measurement occasions and larger samples should examine within-person developmental processes using the RI-CLPM. A further limitation is that the two lag intervals are unequal: W1 to W6 spans 5 weeks of standard flipped instruction, whereas W6 to W15 spans 9 weeks of AI-enhanced instruction. Cross-lagged paths across unequal intervals are not directly comparable, and the longer second interval may partly account for the non-significant W6 to W15 paths.

Future research should examine not only the frequency of AI use but also the quality of students' AI interactions, moving beyond simple measures of AI use. Although the AI learning logs were used only to monitor intervention implementation in the present study, they provide an opportunity for future research to investigate prompt sophistication as a potential mechanism linking agentic engagement with self-regulated learning development. Extending the observation period beyond a single semester would help determine whether the observed improvements are sustained over time. Additionally, testing the proposed two-stage developmental model across different subject domains, educational systems, and learner characteristics, including personality traits such as conscientiousness and openness to experience, would clarify the generalizability and boundary conditions of the present findings.

## 6 Conclusion

This study provides rare four-timepoint longitudinal evidence on the co-development of student engagement and self-regulated learning across the transition from standard to AI-enhanced flipped instruction in a Central Asian higher education context. Four distinct engagement trajectories were identified, with agentic engagement demonstrating the strongest and most sustained growth, while all six self-regulated learning strategies followed a coherent dormant-then-activated pattern, remaining stable during the standard flipped phase before increasing during the AI-enhanced phase. The largest gains in self-regulated learning were observed during the W11–W15 interval, spanning the latter stages of AI-enhanced instruction, suggesting that progressively transferring responsibility for learning from instructor to student may be beneficial for supporting self-regulated learning.

The cross-lagged panel analyses further showed that early agentic engagement predicted subsequent metacognitive self-regulation, providing longitudinal evidence that proactive participation may serve as an important precursor to later self-regulatory development. Although the hypothesized mediation through academic achievement was not supported, the findings underscore the complexity of the relationship between self-regulation and conventional knowledge-based assessments.

Overall, the findings suggest a two-stage developmental process in which standard flipped instruction and progressively scaffolded AI integration may each play distinct roles in supporting student engagement and self-regulated learning across an academic semester. Although causal attribution is not possible within the present within-subjects design, the observed trajectories offer a theoretically coherent basis for future experimental investigation of AI-enhanced flipped learning in non-Western higher education contexts.

From a practical perspective, the findings suggest that the educational value of generative AI depends less on simply providing access to AI tools than on designing learning environments that progressively cultivate learner autonomy. The guided, semi-guided, and self-directed instructional sequence implemented in this study provides a practical framework for integrating AI into higher education in ways that support both student engagement and self-regulated learning.

## Data Availability

The raw data supporting the conclusions of this article will be made available by the authors, without undue reservation.
