# Supplementary material for: Student engagement and self-regulated learning across standard and AI-enhanced flipped learning: a within-subjects longitudinal study
Source: Front Psychol. 2026 Aug 19;17:1936442. doi: 10.3389/fpsyg.2026.1936442 (PMC13533682; doi:10.3389/fpsyg.2026.1936442)
Supplement: Supplementary file 1 [file Supplementary_File_1.docx]

**Supplementary Table S1**

**Baseline Attrition Analysis Comparing Retained and Excluded Participants**

| **Variable** | **Retained (n = 211) M (SD) or %** | **Excluded (n = 95) M (SD) or %** | **t or χ²** | **df** | **p** | **d** |
| --- | --- | --- | --- | --- | --- | --- |
| **Engagement subscales** |  |  |  |  |  |  |
| Behavioral Engagement | 4.13 (0.64) | 3.84 (0.53) | 3.85 | 285.4 | < .001 | **0.50** |
| Emotional Engagement | 4.20 (0.72) | 3.94 (0.73) | 2.31 | 191.2 | .022 | **0.36** |
| Cognitive Engagement | 3.87 (0.67) | 3.75 (0.66) | 1.39 | 188.4 | .166 | 0.17 |
| Agentic Engagement | 2.75 (0.96) | 2.50 (1.02) | 1.78 | 176.3 | .307 | 0.25 |
| **SRL subscales** |  |  |  |  |  |  |
| Rehearsal | 3.65 (0.71) | 3.52 (0.68) | 1.44 | 192.6 | .151 | 0.19 |
| Elaboration | 3.79 (0.67) | 3.71 (0.63) | 0.96 | 195.8 | .340 | 0.12 |
| Organisation | 3.57 (0.80) | 3.48 (0.76) | 0.89 | 193.1 | .375 | 0.11 |
| Critical Thinking | 3.73 (0.70) | 3.65 (0.68) | 0.89 | 194.3 | .376 | 0.12 |
| Metacog. Self-Regulation | 3.48 (0.72) | 3.38 (0.68) | 1.10 | 190.7 | .272 | 0.14 |
| Help-Seeking | 3.23 (1.03) | 2.92 (0.96) | 2.37 | 194.5 | .019 | **0.30** |
| **Academic achievement** |  |  |  |  |  |  |
| Achievement (W1, 0–15) | 9.47 (2.82) | 9.60 (2.52) | −0.37 | 188.9 | .714 | −0.05 |
| **Demographic characteristics** |  |  |  |  |  |  |
| Age, years | 20.1 (1.5) | 19.8 (1.6) | 1.41 | 177.2 | .160 | 0.19 |
| GPA (0–4 scale) | 3.06 (0.54) | 3.01 (0.58) | 0.68 | 181.3 | .499 | 0.09 |
| Female, % | 88.6 | 86.3 | χ²(1) = 0.37 | — | .545 | — |
| Year of study, % |  |  |  |  |  |  |
| First year | 19.9 | 22.1 | χ²(3) = 1.24 | — | .743 | — |
| Second year | 59.7 | 57.9 |  |  |  |  |
| Third year | 11.8 | 12.6 |  |  |  |  |
| Fourth year or above | 8.5 | 7.4 |  |  |  |  |

*Note.* Retained participants contributed data from at least 8 of the 10 study participation events, whereas excluded participants contributed data from fewer than 8 events. Independent-samples t-tests with Welch's correction (unequal variances assumed) were used for continuous variables. Chi-square tests were used for categorical variables. Cohen's d was computed as the standardised mean difference between retained and excluded groups; d values are not reported for categorical variables. Effect sizes in bold indicate statistically significant group differences (p < .05). SRL = self-regulated learning; GPA = grade point average; Metacog. = Metacognitive. Engagement and SRL subscales were rated on a 1–5 Likert scale. Achievement scores reflect the 15-item multiple-choice knowledge test administered at Week 1.

**Supplementary Table S2**

**Factor Loadings, Model Fit, and Validity Indices for the Engagement Scale (N = 211)**

| **Factor / Item** | **λ (STDYX)** | **SE** | **p** |
| --- | --- | --- | --- |
| **Emotional Engagement** | EMO1 | .882 | .030 |
|  | EMO2 | .937 | .025 |
|  | EMO3 | .924 | .024 |
| **Behavioral Engagement** | BEH1 | .797 | .032 |
|  | BEH2 | .816 | .032 |
|  | BEH3 | .788 | .032 |
|  | BEH4 | .827 | .031 |
| **Cognitive Engagement** | COG1 | .623 | .039 |
|  | COG2 | .744 | .030 |
|  | COG3 | .698 | .035 |
|  | COG4 | .790 | .026 |
|  | COG5 | .736 | .031 |
|  | COG6 | .808 | .026 |
|  | COG7 | .834 | .023 |
|  | COG8 | .833 | .029 |
| **Agentic Engagement** | AGEN1 | .803 | .027 |
|  | AGEN2 | .876 | .019 |
|  | AGEN3 | .847 | .025 |
|  | AGEN4 | .859 | .021 |
|  | AGEN5 | .812 | .025 |
| **Fit Index** | **Value** | | |
| CFI | .968 |  |  |
| TLI | .963 |  |  |
| RMSEA | .077 [90% CI: .038, .107] |  |  |
| SRMR | .055 |  |  |

**Convergent and Discriminant Validity**

| **Subscale** | **EMO** | **BEH** | **COG** | **AGEN** | **CR** | **AVE** | **√AVE** |
| --- | --- | --- | --- | --- | --- | --- | --- |
| **EMO** | **[0.915]** |  |  |  | .939 | .837 | .915 |
| **BEH** | 0.592 | **[0.807]** |  |  | .882 | .651 | .807 |
| **COG** | 0.402 | 0.611 | **[0.761]** |  | .916 | .580 | .761 |
| **AGEN** | 0.273 | 0.500 | 0.417 | **[0.840]** | .923 | .705 | .840 |

*Note.* Factor loadings (λ) are STDYX-standardized estimates from a four-factor CFA estimated in Mplus 8.3 using WLSMV (THETA parameterization). All factor loadings were statistically significant (*p* < .001). Diagonal values in brackets represent √AVE; off-diagonal values are latent factor correlations. Composite reliability (CR) ranged from .882 to .939 and AVE ranged from .580 to .837, supporting convergent validity. Discriminant validity was supported according to the Fornell–Larcker criterion.

**Supplementary Table S3**

**Factor Loadings, Model Fit, and Validity Indices for the MSLQ-M (N = 211)**

| **Factor / Item** | **λ (STDYX)** | **SE** | **p** |  |
| --- | --- | --- | --- | --- |
| **Rehearsal** | REHEA1 | .533 | .057 |  |
|  | REHEA2 | .560 | .053 |  |
|  | REHEA3 | .679 | .048 |  |
|  | REHEA4 | .659 | .057 |  |
| **Elaboration** | ELAB1 | .589 | .046 |  |
|  | ELAB2 | .669 | .039 |  |
|  | ELAB3 | .829 | .026 |  |
|  | ELAB4 | .619 | .044 |  |
|  | ELAB5 | .811 | .030 |  |
|  | ELAB6 | .605 | .041 |  |
| **Organisation** | ORG1 | .607 | .046 |  |
|  | ORG2 | .713 | .039 |  |
|  | ORG3 | .582 | .046 |  |
|  | ORG4 | .723 | .036 |  |
| **Critical Thinking** | CT1 | .726 | .036 |  |
|  | CT2 | .633 | .036 |  |
|  | CT3 | .787 | .029 |  |
|  | CT4 | .696 | .031 |  |
|  | CT5 | .722 | .037 |  |
| **Metacog. Self-Regulation** | METASR1 | .657 | .040 |  |
|  | METASR2 | .691 | .039 |  |
|  | METASR3 | .555 | .048 |  |
|  | METASR4 | .556 | .045 |  |
|  | METASR5 | .626 | .042 |  |
|  | METASR6 | .741 | .032 |  |
|  | METASR7 | .619 | .037 |  |
|  | METASR8 | .755 | .032 |  |
|  | METASR9 | .711 | .036 |  |
|  | METASR10 | .690 | .036 |  |
|  | METASR11 | .765 | .031 |  |
|  | METASR12 | .720 | .036 |  |
| **Help-Seeking** | HELPS1 | .888 | .032 |  |
|  | HELPS2 | .792 | .038 |  |
|  | HELPS3 | .798 | .032 |  |
|  | HELPS4 | .744 | .039 |  |
| **Fit Index Value** | | | | |
| **CFI** | .945 |  |  |  |
| **TLI** | .940 |  |  |  |
| **RMSEA** | .057 [90% CI: .046, .068] |  |  |  |
| **SRMR** | .062 |  |  |  |
|  |  |  |  |  |

**Convergent and Discriminant Validity**

| **Subscale** | **REHEA** | **ELAB** | **ORG** | **CT** | **METASR** | **HELPS** | **CR** | **AVE** | **√AVE** |
| --- | --- | --- | --- | --- | --- | --- | --- | --- | --- |
| **REHEA** | **[0.611]** |  |  |  |  |  | .702 | .373† | .611 |
| **ELAB** | 0.719* | **[0.694]** |  |  |  |  | .845 | .481† | .694 |
| **ORG** | 0.736* | 0.836* | **[0.659]** |  |  |  | .753 | .435† | .659 |
| **CT** | 0.536 | 0.818* | 0.802* | **[0.715]** |  |  | .838 | .511 | .715 |
| **METASR** | 0.721* | 0.752* | 0.755* | 0.839* | **[0.677]** |  | .910 | .459† | .677 |
| **HELPS** | 0.293 | 0.487 | 0.482 | 0.485 | 0.620 | **[0.807]** | .882 | .652 | .807 |
|  |  |  |  |  |  |  |  |  |  |

*Note.* Factor loadings (λ) are STDYX-standardized estimates from a six-factor CFA estimated in Mplus 8.3 using WLSMV with PARAMETERIZATION = THETA. All loadings are statistically significant (p < .001). SE = standard error. Loadings ≥ .70 are shown in bold. CR = Composite Reliability; AVE = Average Variance Extracted. † AVE values below .50 indicate limited convergent validity according to conventional criteria. However, composite reliability (CR) exceeded .70 for all subscales, indicating acceptable internal consistency. Diagonal elements in brackets show √AVE values. Off-diagonal elements show factor correlations; values marked with an asterisk (*) indicate Fornell–Larcker violations where the inter-factor correlation exceeds the √AVE of both factors. Several Fornell–Larcker criterion violations were observed among the conceptually related SRL subscales, indicating substantial overlap between Elaboration, Organisation, Critical Thinking, and Metacognitive Self-Regulation. Discriminant validity among these constructs should therefore be interpreted with appropriate caution.

**Supplementary Table S4**

**Longitudinal Measurement Invariance Results for the Engagement Scale Across Four Measurement Occasions**

| **Model** | **Δdf** | **DIFFTEST χ²** | **CFI** | **TLI** | **RMSEA** | **ΔCFI** | **ΔRMSEA** | **Conclusion** |
| --- | --- | --- | --- | --- | --- | --- | --- | --- |
| **Configural** | — | — | .905 | .896 | .061 | — | — | Supported |
| **Metric** | 48 | 127.013 | .912 | .904 | .058 | +.007 | −.003 | Supported |
| **Scalar** | 126 | 1025.023 | .886 | .881 | .065 | −.026 | +.007 | Not supported |

*Note.* Models were estimated in Mplus 8.3 using WLSMV estimation with THETA parameterization. Nested models were compared using the DIFFTEST procedure. Metric invariance was supported according to ΔCFI and ΔRMSEA criteria (Cheung & Rensvold, 2002; Chen, 2007), whereas scalar invariance was not supported because the decrease in CFI exceeded the recommended threshold. Consequently, subsequent longitudinal analyses were interpreted primarily on the basis of metric invariance.

**Supplementary Table S5**

**Longitudinal Measurement Invariance Results for the MSLQ-M Subscales Across Four Measurement Occasions**

| **Subscale** | **Config CFI** | **Config RMSEA** | **Metric CFI** | **Metric RMSEA** | **ΔCFI** | **ΔRMSEA** | **Conclusion** |
| --- | --- | --- | --- | --- | --- | --- | --- |
| **Rehearsal** | .962 | .073 | .962 | .073 | .000 | .000 | Supported |
| **Elaboration** | .885 | .097 | .885 | .094 | .000 | −.003 | Supported |
| **Organisation** | .953 | .086 | .962 | .073 | +.009 | −.013 | Supported |
| **Crit. Thinking** | .868 | .116 | .896 | .099 | +.028 | −.017 | Supported* |
| **Metacog. SR** | .835 | .082 | .848 | .078 | +.013 | −.004 | Supported* |
| **Help-Seeking** | .953 | .086 | .962 | .073 | +.009 | −.013 | Supported |
|  |  |  |  |  |  |  |  |

**Note.** Models were estimated separately for each MSLQ-M subscale in Mplus 8.3 using WLSMV estimation with THETA parameterization (Little, 2013). Configural and metric invariance models are reported. Metric invariance was evaluated using changes in approximate fit indices (Cheung & Rensvold, 2002; Chen, 2007). Metric invariance was considered supported when changes in fit indices met recommended criteria (ΔCFI ≥ −.010 and ΔRMSEA ≤ .015). Subscales marked with an asterisk (*) satisfied these criteria but exhibited comparatively weak absolute fit for the configural model; therefore, these results should be interpreted with appropriate caution. Config = Configural model; Metacog. SR = Metacognitive Self-Regulation.

**Supplementary Table S6**

**Item-Level Statistics for the 15-Item Achievement Test Across Three Administrations**

| **No.** | **Topic** | **Key** | Week 1 | | Week 6 | | Week 15 | | **Psychometric**  **Flag** |
| --- | --- | --- | --- | --- | --- | --- | --- | --- | --- |
|  |  |  | **Difficulty** | **Discrim.** | **Difficulty** | **Discrim.** | **Difficulty** | **Discrim.** |  |
| 1 | Behaviorism | A | 0.746 | 0.473 | 0.869 | 0.447 | 0.865 | 0.613 |  |
| 2 | Universal Grammar | D | 0.648 | 0.536 | 0.672 | 0.507 | 0.805 | 0.592 |  |
| 3 | Cognitivism | B | 0.402 | 0.259 | 0.537 | 0.259 | 0.553 | 0.323 |  |
| 4 | Constructivism | C | 0.549 | 0.514 | 0.722 | 0.407 | 0.786 | 0.567 |  |
| 5 | Grammar Rules | D | 0.773 | 0.387 | 0.811 | 0.424 | 0.879 | 0.469 |  |
| 6 | Language Aptitude | A | 0.284 | 0.249 | 0.444 | 0.411 | 0.572 | 0.354 |  |
| 7 | Input Modality | B | 0.890 | 0.297 | 0.915 | 0.392 | 0.930 | 0.515 |  |
| 8 | Learning Strategies | C | 0.773 | 0.327 | 0.826 | 0.326 | 0.888 | 0.410 |  |
| 9 | Language Transfer | B | 0.542 | 0.474 | 0.618 | 0.518 | 0.777 | 0.521 |  |
| 10 | Syntax | A | 0.375 | -0.045 | 0.344 | -0.113 | 0.274 | -0.045 | **NEG†** |
| 11 | Cultural Competence | A | 0.299 | 0.105 | 0.290 | -0.026 | 0.419 | 0.014 | **LOW†** |
| 12 | Brain Lateralization | B | 0.769 | 0.360 | 0.803 | 0.344 | 0.907 | 0.363 |  |
| 13 | True/False item | A | 0.932 | 0.135 | 0.950 | 0.244 | 0.944 | 0.152 | **CEILING†** |
| 14 | True/False item | A | 0.852 | 0.187 | 0.780 | 0.202 | 0.860 | 0.189 |  |
| 15 | True/False item | B | 0.727 | 0.354 | 0.768 | 0.442 | 0.870 | 0.426 |  |
| **Test Reliability (KR-20)** | | | .703 | — | .702 | — | .740 | — |  |

*Note.* Difficulty = proportion of students answering correctly (higher values indicate easier items; range = 0–1). Discrim. = corrected item–total correlation (point-biserial), with higher values indicating better item discrimination. KR-20 = Kuder–Richardson Formula 20 reliability coefficient. Key = correct response option. Sample sizes were N = 264 (Week 1), 259 (Week 6), and 215 (Week 15). **† Flagged items:** Item 10 demonstrated negative discrimination across all three administrations. Item 11 showed consistently low discrimination, including one negative value at Week 6. Item 13 exhibited ceiling-level difficulty (proportion correct > .93 across all administrations), limiting its contribution to score variance. These items were retained to preserve an identical test form across measurement occasions and facilitate direct longitudinal comparisons; therefore, their psychometric limitations should be considered when interpreting the achievement results.
